# Complex role of microRNAs in HTLV-1 infections

**DOI:** 10.3389/fgene.2012.00295

**Published:** 2012-12-17

**Authors:** Gavin C. Sampey, Rachel Van Duyne, Robert Currer, Ravi Das, Aarthi Narayanan, Fatah Kashanchi

**Affiliations:** ^1^National Center for Biodefense and Infectious Diseases, School of Systems Biology, George Mason UniversityManassas, VA, USA; ^2^Department of Microbiology, Immunology, and Tropical Medicine, The George Washington University Medical CenterWashington, DC, USA

**Keywords:** HTLV-1, ATL, HAM/TSP, miRNA, Tax, RNAi, chromatin, NF-κB

## Abstract

Human T-lymphotropic virus 1 (HTLV-1) was the first human retrovirus to be discovered and is the causative agent of adult T-cell leukemia/lymphoma (ATL) and the neurodegenerative disease HTLV-1-associated myelopathy/tropical spastic paraparesis (HAM/TSP). The importance of microRNA (miRNA) in the replicative cycle of several other viruses, as well as in the progression of associated pathologies, has been well established in the past decade. Moreover, involvement of miRNA alteration in the HTLV-1 life cycle, and in the progression of its related oncogenic and neurodegenerative diseases, has recently come to light. Several HTLV-1 derived proteins alter transcription factor functionalities, interact with chromatin remodelers, or manipulate components of the RNA interference (RNAi) machinery, thereby establishing various routes by which miRNA expression can be up- or down-regulated in the host cell. Furthermore, the mechanism of action through which dysregulation of host miRNAs affects HTLV-1 infected cells can vary substantially and include mRNA silencing via the RNA-induced silencing complex (RISC), transcriptional gene silencing, inhibition of RNAi components, and chromatin remodeling. These miRNA-induced changes can lead to increased cell survival, invasiveness, proliferation, and differentiation, as well as allow for viral latency. While many recent studies have successfully implicated miRNAs in the life cycle and pathogenesis of HTLV-1 infections, there are still significant outstanding questions to be addressed. Here we will review recent discoveries elucidating HTLV-1 mediated manipulation of host cell miRNA profiles and examine the impact on pathogenesis, as well as explore future lines of inquiry that could increase understanding in this field of study.

## Introduction

Human T-lymphotropic virus 1 (HTLV-1) was discovered in the early 1980s by two-independent groups working in the United States (Poiesz et al., 1980, 1981) and Japan (Yoshida et al., 1982). It is a complex retrovirus and a member of the Deltaretrovirus genus. Although there are currently four known types of HTLV, HTLV-1 is by far the most pathogenic of the group and has the distinction of being the first oncogenic retrovirus discovered in humans (Mahieux and Gessain, 2007). It infects an estimated 15–20 million people worldwide and has been implicated as the causative agent in a number of disease conditions, most notably adult T-cell leukemia/lymphoma (ATL) and HTLV-1-associated myelopathy/tropical spastic paraparesis (HAM/TSP). HAM/TSP was first described in 1969 over a decade prior to the discovery of HTLV-1(Mani et al., 1969). It presents with neuro-inflammatory symptoms and incomplete paralysis of the limbs, although development of this condition among HTLV-1 infected individuals has been estimated at only 0.25% (Mani et al., 1969; Kaplan et al., 1990; Kfoury et al., 2012). ATL was first characterized by the work of Uchiyama and colleagues in 1977 (Uchiyama et al., 1977; Gallo, 2011; Kfoury et al., 2012). Over the lifespan of an HTLV-1 infected patient, the risk of developing ATL is 2.6–4.5% (Tokudome et al., 1989) and the resulting cancer has an aggressive disease course that is highly resistant to current chemotherapy treatments (Tsukasaki et al., 2007, 2009). Due to the devastating nature of the diseases that develop in patients infected with this pathogen and the lack of effective treatments, it is critical to better understand the underlying molecular mechanisms that induce these conditions in order to develop targeted therapeutics.

The molecular mechanisms by which viruses can progress through their life cycle and affect disease states in the host organism are diverse and our understanding of them is constantly evolving. In the case of HTLV-1, the viral protein Tax has been studied in depth as a both a key component for viral replication and in the oncogenic transformation of infected cells (Jeang et al., 1988; Pozzatti et al., 1990; Tanaka et al., 1990; Zhao and Giam, 1992; Baranger et al., 1995; Grossman et al., 1995). In addition to viral proteins, microRNAs (miRNAs) have also been found to play a key role in viral life cycles and related disease progression. Over the past decade, it has been demonstrated that several relatively large DNA viruses, such as the Epstein-Barr virus (EBV), can alter host miRNA profiles and actually encode their own viral miRNAs in order to effect changes to host cells that benefit the virus (Pfeffer et al., 2004; Cameron et al., 2008; Gatto et al., 2008). More recently, it has even been shown that other retroviruses, such as human immunodeficiency virus (HIV-1) and bovine leukemia virus (BLV), can effect similar changes to host miRNAs and also encode several viral miRNAs despite their small genome size (Klase et al., 2007; Houzet et al., 2008; Ouellet et al., 2008; Klase et al., 2009; Althaus et al., 2012; Kincaid et al., 2012; Schopman et al., 2012). Moreover, several studies have also demonstrated that HTLV-1 alters the profile of host miRNAs (Bellon et al., 2009; Rahman et al., 2012). Examining these findings regarding HTLV-1 and miRNA in connection with the recent discoveries of miRNA manipulation by other retroviruses demonstrates the importance of the RNA interference (RNAi) machinery to these related viral infections.

In this review we will examine recent studies that demonstrate the manipulation of the host cellular miRNA profile by HTLV-1 and its relationship to the manifestation of the oncogenic and neurodegenerative diseases associated with this retrovirus. We will further examine potential mechanisms of action that may be responsible for this alteration of miRNA expression and explore future lines of inquiry in this novel field of study. The findings detailed here have significantly transformed our understanding of the life cycle of the HTLV-1 virus and provide new avenues for scientific exploration and therapeutic intervention against this pathogen.

## MicroRNA and the RNAi molecular machinery

### MicroRNA biogenesis and the RNAi molecular machinery

The biogenesis of miRNAs has been very well characterized and is described in multiple articles (Faller and Guo, 2008; Chua et al., 2009; Perron and Provost, 2009; Winter et al., 2009; Ying and Lin, 2009; Van Wynsberghe et al., 2011; Yang and Lai, 2011). MicroRNAs are genome encoded RNA hairpin structures that are usually transcribed by RNA polymerase II (Pol II) as primary transcripts (pri-miRNA) of up to several kilobases in length. The pri-miRNA is produced with a 5′ 7-methyl guanosine (m7G) cap, a 3′ poly-A tail and a structured body with one or more hairpin structures approximately 80 nucleotides long (Perron and Provost, 2008). This pri-miRNA is processed in the nucleus by the RNase III enzyme Drosha in conjunction with its associated double-stranded RNA-binding protein, DiGeorge syndrome critical region 8 (DGCR8) (Lee et al., 2003; Cai et al., 2004; Denli et al., 2004; Han et al., 2004). Drosha cleaves the miRNA at about 22 base pairs down-stream of the stem-loop structure to generate an approximately 60 nucleotide long pre-miRNA with a two nucleotide 3′ overhang. The two nucleotide 3′ overhang in the pre-miRNA is recognized by the exportin-5/Ran GTP complex which then facilitates pre-miRNA export out of the nucleus (Yi et al., 2003; Kim, 2004). In the cytoplasm, the pre-miRNA is bound by a second RNase III enzyme, Dicer that cleaves the RNA about two helical turns into the hairpin and degrades the terminal loop structure (Vermeulen et al., 2005; Macrae et al., 2006; Zhang and Zeng, 2010). Dicer acts in association with the TAR binding protein (TRBP) (Chendrimada et al., 2005; Haase et al., 2005) and generates a miRNA duplex of approximately 22 nucleotides with a two nucleotide overhang at the 3′ ends of both strands.

For post-transcriptional silencing, one strand of the miRNA duplex, the “passenger strand,” is degraded while the other, the “guide strand,” is incorporated into the RNA-induced silencing complex (RISC) (Kawamata and Tomari, 2010). The strand that is less stably base-paired at the 5′ end of the duplex is usually the guide strand (Schwarz et al., 2003). The catalytic components of RISC are the Argonaute proteins (Ago 1–4), of which Ago2 has been shown to have endonuclease activity and can cleave target mRNAs that show complementarity to the guide strand. The RISC complex and the associated miRNA were first found to bind to the 3′ UTR region of the target mRNAs, but subsequent studies found targeting of the 5′ UTR and coding regions as well (Easow et al., 2007; Lytle et al., 2007; Orom et al., 2008; Rigoutsos, 2009; Hafner et al., 2010). Nucleotides 2–7 of the miRNA, called “the seed,” play an important role in the positioning of the RISC complex and the associated miRNA on the target mRNA (Parker et al., 2006, 2009). The degree of complementarity between the target mRNA and the effector miRNA is a determining factor that decides if the target mRNA is degraded or if it is translationally repressed. Extensive complementarity between the target and miRNA will result in mRNA degradation. However, a low level of complementarity will result in translational repression. In mammalian cells, it has been shown that miRNA driven destabilization and degradation of target mRNAs is the predominant route of subsequent protein level suppression (Guo et al., 2010). The mRNA-RISC complex is transported to cytoplasmic structures that contain RNA remodeling components and no translational machinery, known as the processing bodies (P-bodies) and stress granules (SGs) (Liu et al., 2005; Leung et al., 2006). However, P-bodies and SGs are not thought to be a requirement for miRNA-mediated repression (Chu and Rana, 2006).

In addition to post-transcriptional processing, RNA mediated silencing can also operate at the chromatin level to regulate gene expression. MicroRNAs can associate with the RNA-induce transcriptional silencing (RITS) complex and be guided to complementary regions in the chromosomal DNA (Zofall and Grewal, 2006; Buhler and Moazed, 2007). In mammals, it has been found that subunits of the RITS complex include Ago1, Ago2, and TAR binding protein 2 (TRBP2) (Janowski et al., 2006; Kim et al., 2006; Ahlenstiel et al., 2012). In addition to these subunits of the RITS complex, association of Pol II with an unphosphorylated C-terminal domain (CTD) is requisite for association to the complementary chromosomal DNA (Kim et al., 2006). Following association with such genomic regions, the RITS complex recruits factors, such as histone modifying enzymes, which alter the chromatin structure and induce transcriptional gene silencing (TGS) (Verdel et al., 2004; Buhler and Moazed, 2007).

While the above-mentioned mechanism is the most commonly observed processing mechanism for generation of miRNAs, several alternate pathways have also been postulated based largely on deep sequencing studies. These studies offer interesting alternatives to the conventional pathway; however, the data supporting them are largely preliminary and require more extensive validation. A recent review by Yang et al. describes the various alternate mechanisms for miRNA production (Yang and Lai, 2011).

### Virally encoded miRNAs

The crucial role of miRNAs in gene regulation makes them an obvious target for viruses to hijack in order to regulate viral and host gene expression. Thus, there are significant advantages for viruses that can manipulate host miRNA profiles and/or exploit the RNAi machinery in order to alter host and viral gene regulation. Furthermore, it has been demonstrated that viruses themselves generate miRNAs. Unlike viral proteins, miRNAs are not antigenic as they can avoid the INF/PKR-induced pathway, which is normally triggered by double stranded RNA (dsRNA) of at least 45-bp in length (Botos et al., 2009; Umbach and Cullen, 2009). Additionally, viral miRNAs are able to successfully down-regulate the expression of host gene products that harbor anti-viral functionalities. Moreover, their small space requirement of around 200-bp of the viral genome is of significant advantage given the tight constraints on viral genome size (Umbach and Cullen, 2009). Since the original discovery of miRNAs in EBV (Pfeffer et al., 2004; Kok et al., 2010), approximately 436 mature viral miRNAs have been identified across all viruses examined and listed in the miRNA repository miRBase (Griffiths-Jones et al., 2006, 2008; Kozomara and Griffiths-Jones, 2011).

Viral miRNAs generally have been found to follow the same biogenesis pathways as cellular miRNA, although an alternate mechanism of generation utilizing cellular RNA Polymerase III (Pol III) has been observed (Bogerd et al., 2010; Kincaid et al., 2012). Interestingly, the alternate use of Pol III to generate a viral miRNA was observed in a HTLV-1 related deltaretrovirus, BLV. Various techniques for the detection of viral miRNA in an infected cell have been employed, most of which begins with bioinformatics analyses to identify stem-loop structures matching pre-miRNA. This is followed by cDNA cloning and high-throughput sequencing of large numbers of the resultant clones (Sullivan and Ganem, 2005; Barth et al., 2008). These clones are subjected to Northern blot analyses with total cellular RNA, which provides additional confirmation. Massively parallel deep sequencing is another widely used method for the detection of viral miRNA. The highly sensitive SOLiD™ 3 Plus System was used to analyze viral RNA accumulation in HIV-1-infected T lymphocytes. This method detected numerous small RNAs that correspond to the HIV-1 RNA genome (Schopman et al., 2012). Additionally, targeted enrichment of viral small non-coding RNAs has also been used to boost the levels of the target viral miRNAs over 100-fold and verify HIV-1 encoded miRNAs from infected lymphocytes (Althaus et al., 2012). The findings from these aforementioned studies supplant previous experiments which failed to detect miRNAs encoded in retroviruses (Lin and Cullen, 2007). The primary improvement with these more recent sequencing studies that positively identified HIV-1 derived miRNAs is the depth of clones sequenced or enrichment of target clones, which leads to a greater statistical power to detect low abundance RNAs. While no such deep sequencing or targeted enrichment studies have yet been performed with HTLV-1, the positive findings of numerous virally encoded miRNAs from HIV-1 and other retroviruses demonstrate the high probability that the HTLV-1 genome encodes viral miRNAs.

### Viral manipulation of host cell miRNA regulation

Beyond the generation of virally encoded miRNAs, infecting viruses can also alter the expression profiles of the host cell miRNAs. Manipulation of cellular miRNAs represents a key mechanism of viral dysregulation of cellular stasis, as host cell miRNAs, including those specific to hematopoietic cells, have been found to significantly affect cellular proliferation and differentiation, as well as the immune response of mammals (Chen et al., 2004; Fazi et al., 2005; Cobb et al., 2006; Li et al., 2007; Loffler et al., 2007; Ivanovska et al., 2008; Johnnidis et al., 2008; Carissimi et al., 2009; Faraoni et al., 2009; Huang et al., 2009; Lal et al., 2009; Curtale et al., 2010). Thus, an infecting virus can induce a range of host responses, such as resistance to apoptosis, an increase in cellular proliferation, or alteration of transcriptional regulation, to their benefit. Furthermore, some human miRNAs have also been shown to directly target human endogenous retroviruses (HERVs) and invading exogenous retroviruses (IERVs) including HTLV-1 (Hakim et al., 2008). In the case of HIV-1, a cluster of host miRNAs that are up-regulated in resting CD4+ T-cells has been shown to target the 3′-region of the HIV-1 transcript which contributes to latency in this cellular population (Huang et al., 2007). Therefore, the viral manipulation of miRNA levels also can affect either evasion of innate host immune response through down-regulation of anti-viral miRNAs, or establishment of a latently infected cell population through co-opting these miRNAs. Indeed, alteration of host miRNA transcription levels has been detected in other virally infected cells, such as those from EBV infected patients (Cameron et al., 2008; Gatto et al., 2008). One such host miRNA up-regulated by EBV, miR-155, also was shown to aid in viral latency by suppressing NF-κB signaling (Lu et al., 2008). More recently, one study showed the hematopoietic specific miRNAs, including miR-223, miR-181a, miR-150, miR-142.3p, and miR-155, were dysregulated in both *in vitro* and *in vivo* samples of HTLV-1 infected cells (Bellon et al., 2009). This dysregulation was found to favor differentiation of infected cell lines as expression levels of different endogenous miRNAs has been found to vary among cell types of different lineages (Merkerova et al., 2008). Also, miR-155, miR-125a, miR-132, and miR-146, which are regulatory components of the innate immune response, were found to be dysregulated. Lastly, the response of two of these dysregulated miRNAs, specifically miR-150 and miR-223, diverged between the infected *in vitro* cell line and isolated patient cells (Bellon et al., 2009). This is only one example in which host miRNA profiles were shown to be altered in relation to HTLV-1 infection and others will be described in more detail later in the review.

## Epigenetic regulation and chromatin remodeling to alter host miRNA profiles

One of the strategies utilized by retroviruses to regulate both viral and cellular transcription, including the transcription of miRNA genes, is through the modulation of higher order chromatin structure, often through manipulating epigenetic markers. This is important for retroviruses in particular, due to the need for the provirus to integrate into the host genome, the efficiency of which is governed by the presence of either a heterochromatic or euchromatic state. HLTV-1 has been well documented to interact with a variety of host cellular chromatin modifying enzymes, such as histone acetyltransferases (HATs), histone methyltransferases (HMTs), and ATP-dependent chromatin remodelers, all of which are manipulated by viral proteins to ensure a favorable transcriptional state. The interaction of the HTLV-1 viral protein Tax with these cellular enzymes, as well as transcription factors, results in the activation of the viral promoter and the production of viral transcripts. Similarly, the viral manipulation of chromatin remodeling enzymes could also alter transcription of cellular genes including those encoding miRNAs. Additionally, due to the ability of Tax to manipulate chromatin structure and the innate host cellular defense mechanism of RNAi to regulate pathogen gene expression, there is most likely interplay between these two competing mechanisms. Here, we also introduce the reciprocal interaction of HTLV-1-induced epigenetic regulation and chromatin remodeling with the host cellular RNAi response.

### HTLV-1 interaction with HATs

**Figure 1:**
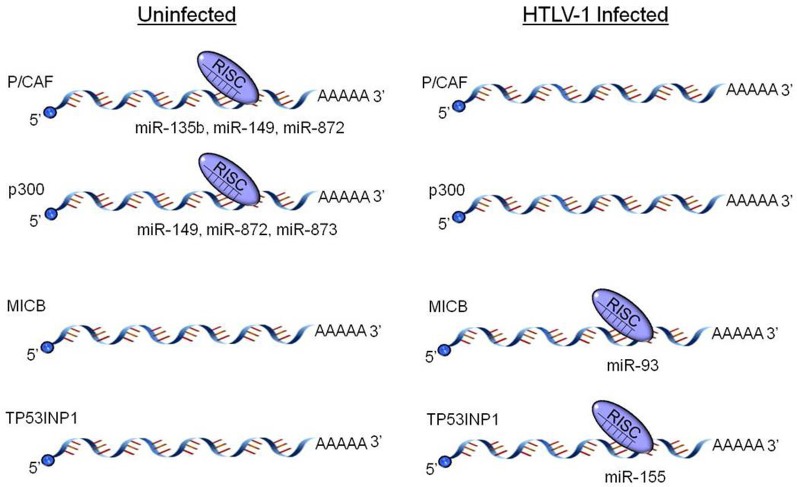
**HTLV-1 dysregulation of host miRNA profiles.** HTLV-1 infected cells selectively up- and down-regulate numerous host cell miRNAs. Specifically, several miRNAs targeting the HATs P/CAF and p300 are decreased in HTLV-1 and Tax transfected cells, while other miRNAs targeting pro-apoptotic (TP53INP1) and immunomodulating genes (MICB) are up-regulated. The dysregulated miRNAs and target proteins detailed here represent only a small fraction of the cellular pathways manipulated by the HTLV-1 virus.

The viral transactivator, Tax, activates the HTLV-1 viral promoter within the long terminal repeat (LTR) by interacting with Tax-responsive elements (TREs) in the U3 region of the LTR. Instead of binding to DNA directly, Tax induces transcription of TREs, catalyzes post-translational modifications (PTMs) of TRE-binding factors, and forms complexes with transcription factors. Tax interacts with numerous important transcription factors and cellular kinases but of these CRE Binding Protein (CREB) is key to viral transcription (Caron et al., 1993; Suzuki et al., 1994; Yin et al., 1995, 1998; Clemens et al., 1996; Colgin and Nyborg, 1998; Harrod et al., 1998; Gachon et al., 2000; Nicot et al., 2000; Xiao et al., 2001; Kashanchi and Brady, 2005; Easley et al., 2010). The interaction of Tax with CREB occurs in order to bind to *cis*-acting replication element (CRE) enhancer sequences on the viral promoter, which is a critical step in viral transactivation and in the formation of the pre-initiation complex (Kwok et al., 1996; Giebler et al., 1997; Kashanchi et al., 1998; Wu et al., 2004; Easley et al., 2010). This interaction is also dependent on the presence of the HATs CREB binding protein (CBP), p300, and p300/CBP-associated factor (P/CAF) to activate HTLV-1 gene expression. In addition to Tax interacting with these HATs to directly activate viral transcription, the recruitment of these HATs to the viral LTR promotes the covalent modification of histone tails on adjacent nucleosomes to promote transcriptionally favorable chromatin state. In particular, CBP and p300 can acetylate histones H2A, H2B, H3, and H4, resulting in a conformational change. Additionally, Tax also reduces histone protein transcript levels in HTLV-1 infected cells (Bogenberger and Laybourn, 2008; Easley et al., 2010; Rahman et al., 2012). Beyond directly interacting with and recruiting these HATs to the viral promoter, Tax has also been shown to down-regulate numerous miRNAs that target p300 and P/CAF, thereby boosting overall availability of these HATs (Figure 1) (Rahman et al., 2012). In turn, the up-regulation of p300 and P/CAF will allow for activation of other host genes reliant on these chromatin remodelers including those encoding miRNAs. Furthermore, Tax could also recruit CREB and its associated HATs to other cellular genes, including those encoding miRNAs, which contain sequences homologous to the TREs, and up-regulate their transcription. In fact, Tax mediated up-regulation via CREB signaling pathway activation has been observed with the host gene β-catenin (Tomita et al., 2006).

### HTLV-1 and protein methylation

Histone methylation often occurs as a signal of a repressed chromatin state as lysine residues on exposed histone tails cannot be both acetylated and methylated simultaneously. Indeed, a hallmark of a heterochromatin state is often di- or tri-methylation of H3 at residue K9 (H3K9me2/3). Conversely, histone methylation can also be an indicator of transcriptional activation, such as methylation of H3 at residue K4 (H3K4) (Ooi et al., 2007; Fan et al., 2008; Meissner et al., 2008; Cedar and Bergman, 2009). Tax interacts with the HMT SUV39H1 at the LTR of HTLV-1 infected cells, which subsequently represses Tax transactivation, as well as methylates H3K9, forming a heterochromatinized state (Johnnidis et al., 2006). Additionally, Tax has been shown to interact with SMYD3, an H3K4 methyltransferase, influencing the subcellular localization of Tax as well as enhancing Tax-dependent activation of the NF-κB pathway (Yamamoto et al., 2011). Moreover, the activation of the NF-κB pathway by Tax is a key mechanism by which HTLV-1 can up-regulate several host miRNAs, as will be discussed in detail later. Therefore, Tax association with SMYD3 is important in enabling this mechanism of viral manipulation of the host miRNA profile. Furthermore, the interaction of Tax with these cellular HMTs could also influence the epigenetic state of cellular genes and hence alter the host miRNA profile.

### HTLV-1 and ATP-dependent chromatin remodeling enzymes

While the epigenetic regulation of histones and chromatin by HATs and HMTs can induce conformational changes, more significant nucleosomal alterations require yet another class of chromatin remodelers. ATP-dependent chromatin remodeling complexes (CRCs) are powerful molecular motors that use energy derived from ATP hydrolysis to disrupt histone/DNA interactions, thereby exposing nucleosomal DNA (Chiba et al., 1994; Reyes et al., 1998). Several classes of CRCs have been isolated from eukaryotic cells and all of them contain a related ATPase motor subunit. The most well documented mammalian CRC is the BAF [Brahma-related gene (BRG)/Brahma-associated factor] complexes which are homologous to the yeast SWI/SNF (switching-defective/sucrose non-fermenting) complexes (De La Serna et al., 2006). This complex is composed of one ATPase subunit, either BRG1 or BRM and a multitude of other protein subunits, all of which comprise their own chromatin-remodeling complex which can either activate or repress chromatin remodeling, thereby altering transcription. Our laboratory previously showed that BRG1 is essential for both Tax transactivation and viral replication (Easley et al., 2010). When recruited by Tax, the BRG1-containing CRC, PBAF, is responsible for nucleosomal remodeling at the LTR, specifically, the removal of Nuc-1, promoting active viral transcription. Alternately, Tax could recruit these ATP-dependent CRCs to other locations within the host genome and alter the nucleosomes within the promoter of other genes including those encoding miRNAs.

### Modulation of chromatin remodeling by miRNA with HTLV-1 infection

Chromatin remodeling in the context of a retroviral infection begins when the provirus integrates into the host genome. The efficacy of the integration is based on the chromatin structure at the site, often dictating an active infection (insertion into a euchromatic region) or a latent infection (insertion into a heterochromatic region). It has recently been shown that the RNAi machinery in coordination with endogenous miRNAs or exogenous siRNA can play a role in chromatin structure and reorganization, especially in the context of cancer and viral infections. As will be discussed in detail later, TGS can occur through RNA Pol II in an RNAi-dependent manner by recruitment of epigenetic modifiers (Kato et al., 2005; Merkenschlager and Wilson, 2008). Interestingly, miRNAs originate from promoters that are subject to the local chromatin structure and the miRNAs produced can ultimately affect the native chromatin structure creating a regulatory feed-back loop. In regards to retroviruses, the expression profile of miRNAs is dramatically different in cells infected with HTLV-1 and HIV-1, as compared to their uninfected counterparts. Furthermore, virally encoded miRNAs have been identified as being produced from integrated HIV-1 as well as other RNA viruses and, therefore, it is possible that HTLV-1 encodes as of yet undiscovered viral miRNAs (Ouellet et al., 2008; Klase et al., 2009; Perez et al., 2010; Narayanan et al., 2011; Althaus et al., 2012; Kincaid et al., 2012; Schopman et al., 2012). Indeed, the RNAi pathway is an appropriate target for retroviruses to manipulate in order to regulate proteins, such as transcription factors and chromatin remodelers, that are needed for viral replication or to establish latency.

**Figure 2:**
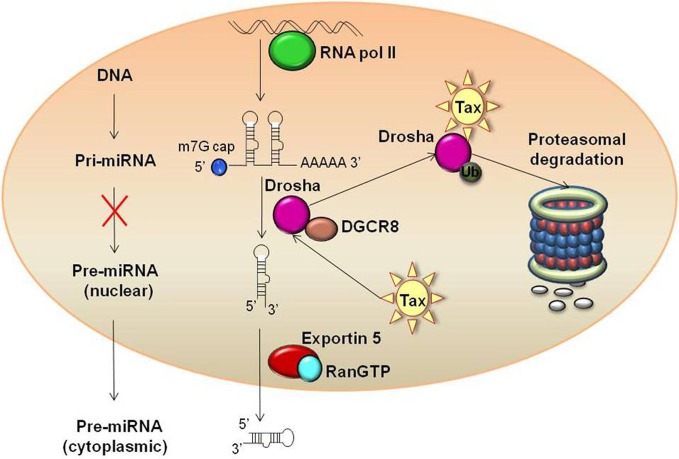
**Tax mediated proteasomal degradation of Drosha.** Tax binds to Drosha and drives subsequent ubiquitination and proteasomal degradation of the protein. This reduction in cellular levels of Drosha in turn leads to global down-regulation of host miRNAs by minimizing the enzymatic machinery to convert Pri-miRNAs to Pre-miRNAs.

A recent study investigated transcription factor profiles as well as profiles of proteins and miRNAs which are Tax-responsive in a stably integrated HTLV-1 LTR T cell clone (Rahman et al., 2012). This study identified that not only does Tax increase the activation of substrates and factors associated with chromatin remodeling, but it down-regulates miRNAs which target factors needed for chromatin remodeling. Specifically, several substrates for HATs and CRCs were up-regulated, including E2F1, GATA, TFIID, nuclear receptors, glucorcorticoid receptors, retinoid X receptors, and vitamin D receptors. Other previously identified HTLV-1 associated transcription factors, such as CREB and Sp1, were also up-regulated. In regards to miRNA profile alteration, miR-135b, miR-149, and miR-872, which specifically target P/CAF, were down-regulated in the presence of Tax, as well as miR-149, miR-872, and miR-873, which target p300 (Figure 1). It is hypothesized that Tax imparts this up-regulation of HATs through suppression of Dicer or Drosha, key RNAi components. This type of suppression has previously been seen in HIV-1, as well as with primate foamy virus type 1 (Bennasser et al., 2005; Lecellier et al., 2005). While a comparable mechanism has yet to be fully elucidated in regards to HTLV-1, potential pathways have been identified. Specifically, recent studies from our lab have shown Tax associates with Drosha and likely targets it for proteasomal degradation (Van Duyne et al., 2012). The mechanism of Tax mediated proteasomal degradation has previously been observed with other cellular proteins including retinoblastoma, and Tax actually boosts the proteolytic activity of the proteasome (Hemelaar et al., 2001; Kehn et al., 2005). This novel mechanism of Drosha suppression reduces the ability to process pri-miRNA into mature miRNAs, thereby presenting a probable mechanism for the global down-regulation of most miRNAs observed with Tax expressing cells (Figure 2) (Rahman et al., 2012).

### Potential transcriptional gene silencing in HTLV-1 infections

Transcriptional gene silencing (TGS) occurring through RNA complementary to gene promoters was first observed and examined in detail in plant species starting around 2000 (Mette et al., 2000). In 2004, studies utilizing *S. pombe* fission yeast first identified several of the components of the RNAi-dependent RITS complex that are required for TGS and postulated a mechanism of action (Noma et al., 2004; Verdel et al., 2004). That same year, mammalian species were shown to carry the same molecular mechanism of gene silencing. Initial evidence of TGS via small ncRNAs in mammals was established using a model system comprised of an elongation factor 1 alpha (EF1α) promoter fused to GFP, which was integrated into the genome of human 293T cells with a feline immunodeficiency virus lentiviral vector. The EF1α-GFP reporter was then silenced by transfection of EF1α promoter targeting siRNA and the silencing mechanism was shown to be induced by DNA methylation (Morris et al., 2004). Another early study showed that induced TGS could be achieved with shRNAs complementary to the promoter region or up to 23 nucleotides down-stream of the transcriptional start site of the RASSF1A tumor suppressor gene. The observed TGS in this experiment also caused low levels of DNA methylation and partial gene silencing in stably transfected HeLa cultures (Castanotto et al., 2005). A relevant comprehensive review of TGS in HIV-1 infections has recently been published (Sampey et al., 2012).

#### Molecular mechanism of transcriptional gene silencing in mammalian cells

While the molecular mechanisms involved in mammalian TGS have still not been clearly defined, a set of studies by Kim et al. helped to elucidate several of the proteins necessary for this mode of gene silencing (Kim et al., 2006). In their studies, mammalian TGS was examined using both transfected siRNA and stably integrated shRNA that targeted either a GFP reporter construct under the control of the *CCR5* promoter or the endogenous *RASSF1A* promoter. Chromatin immunoprecipitation (ChIP) experiments showed enrichment of H3K9me2 at the target promoter of the siRNA, as well as in proximal flanking DNA up to 300-bp down-stream of the target promoter. Furthermore, this boost in the H3K9me2 epigenetic marker was found to increase over a 24 h period post-transfection. Moreover, Ago1 was also enriched at the target promoter and flanking DNA but, unlike the H3K9me2 enrichment, the Ago1 enrichment was transient, peaking at 6 h post-transfection and then rapidly decreasing over the following 18 h. It was also found that the siRNA mediated knockdown of Ago1 disrupted both Ago1 and H3K9me2 enrichment at the target promoter following siRNA transfection indicating the role of Ago1 in recruiting siRNA and/or the necessary HMTs to the promoter. The involvement of Ago1, as well as Ago2, in mammalian TGS was also found to be required for the formation of the RITS complex in other TGS studies (Janowski et al., 2006; Ahlenstiel et al., 2012). The most recent of these studies showed that HIV-1 promoter targeting siRNAs were found to co-localize with RITS-like component Ago1 in the nucleus and Ago2 at the inner nuclear membrane. This work also indicated the involvement of actin in the transport of this RITS-like complex and showed siRNA specificity in this translocation as scrambled siRNA was retained in the cytoplasm (Ahlenstiel et al., 2012).

Beyond Ago1 and Ago2, TRBP2 was also found to be enriched at the target *CCR5* promoter after siRNA transfection. Similar to the Ago1 knockdown, the knockdown of TRBP2 via siRNA also blocked subsequent TGS inhibition of the reporter gene mRNA. These data therefore, clearly demonstrated the mechanistic necessity of TRBP2 in RNAi-induced TGS (Kim et al., 2006). It was also demonstrated that Ago1 was able to co-precipitate with Pol II containing an unphosphorylated CTD after degrading associated RNA, thereby showing a direct protein-protein interaction between these two components. Finally, the histone methyltransferase EZH2, a component of the Polycomb silencing machinery, was also found to be enriched at siRNA targeted endogenous promoters indicating its potential role in the epigenetic modification related to TGS. As EZH2 boosts H3K27me3 levels, this isoform of H3 was also found to be enriched at the targeted promoters (Kim et al., 2006). Furthermore, EZH2 is also able to bind and recruit DNMTs via its homology II domain, thereby providing yet another mechanism by which additional epigenetic silencing may occur (Vire et al., 2006).

Additionally, experiments targeting the endogenous EF1α promoter with nuclear targeted siRNA also showed an increase in the levels of H3K9me2 and H3K27me3. The observed increase in the H3K9me2 was found up to 720-bp down-stream of the targeted siRNA site, demonstrating the spread of the induced epigenetic changes to DNA distal to the target site. Furthermore, a Pol II inhibitor effectively blocked the increase in the TGS associated epigenetic marker, H3K9me2, indicating the requirement for Pol II in siRNA mediated TGS. It was also found that the antisense strand and double stranded siRNA targeting the EF1α promoter also bound to the DNA methyltransferase, DNMT3A. Additional experiments with a HIV-1 LTR reporter cell line further showed that antisense siRNA alone targeting the U3 region of the LTR was sufficient to down-regulate the reporter activity (Weinberg et al., 2006).

#### Endogenously induced TGS in retroviral infections

While numerous studies of TGS have been conducted utilizing exogenous small ncRNAs, such as siRNA and shRNA, very little evidence links endogenously produced ncRNAs to this important gene expression altering pathway. One study depicted the bidirectional transcription of genes as generating competing sense and antisense RNA strands that could alter gene expression by controlling TGS (Morris et al., 2008). In this model, the antisense strand expression could down-regulate the transcription of the complimentary gene through recruitment of Ago1 and the repressive H3K27me3 epigenetic marker to the sense strand promoter. Conversely, reduction of the antisense strand using siRNA minimized negative epigenetic regulation of the sense strand, thereby boosting sense mRNA transcription.

In relation to TGS in retroviral infections, numerous studies have identified antisense transcripts and antisense proteins produced by HIV-1 and HTLV-1 over the past several decades (Miller, 1988; Larocca et al., 1989; Michael et al., 1994; Vanhee-Brossollet et al., 1995; Peeters et al., 1996; Tagieva and Vaquero, 1997; Briquet and Vaquero, 2002; Bentley et al., 2004; Ludwig et al., 2006; Satou et al., 2006; Landry et al., 2007; Yeung et al., 2009; Bansal et al., 2010; Clerc et al., 2011; Lefebvre et al., 2011; Schopman et al., 2012). Building upon this work, it was recently shown that in HIV-1, the U3 region of the 3′ LTR contains a promoter that generates a 2.6 kb antisense RNA (asRNA) with transcriptional termination within the *Env* gene. The promoter of this viral antisense transcript is driven by a NF-κB enhancer site that is responsive to TNFα treatment but not Tat. Furthermore, its over-expression led to repression of HIV-1 replication for up to a month and, conversely, siRNA repression of the asRNA up-regulated HIV-1 replication. While the mechanism by which the HIV-1 asRNA inhibits viral replication was not fully elucidated, it was shown that viral entry and integration were likely not inhibited by asRNA over-expression, but that sense strand expression was inhibited (Kobayashi-Ishihara et al., 2012).

Similarly, HTLV-1 is known to transcribe an asRNA that encodes the viral protein HBZ. The *HBZ* gene has further been shown to effect cells through a bimodal mechanism by which both the protein product and the asRNA transcript have distinct functionalities (Satou et al., 2006). Specifically, while the HBZ protein inhibits Tax-mediated transactivation and viral transcription, the HBZ asRNA causes increased proliferation of T cells. Furthermore, mutational analysis showed that the first stem-loop structure of the HBZ asRNA was required for the observed growth-promoting activity, although initial attempts at detecting miRNAs derived from this structure were unsuccessful. It has also been shown that the vast majority of the HBZ asRNA is retained in the nucleus indicating the asRNA functionality as a ncRNA regulator may be predominant (Rende et al., 2011). Additionally, it has also been shown that the levels of the HBZ asRNA positively correlated with disease severity in patients exhibiting HAM/TSP (Saito et al., 2009). While the studies of the *HBZ* gene to date have attributed the inhibition of viral transcription solely to the protein product, it is possible that the asRNA transcript could also function in an inhibitory fashion similar to that observed in HIV-1. Overall, these findings could demonstrate that the asRNA transcribed by the HTLV-1 provirus could function similarly to the model of competing sense and antisense described earlier. In order to demonstrate such a mechanism in regards to an HTLV-1 infection, though, further studies showing asRNA-induced TGS and concurrent recruitment of Ago1 and/or repressive epigenetic markers are necessary.

## Tax mediated activation of NF-κB to alter host miRNA profiles

**Figure 3:**
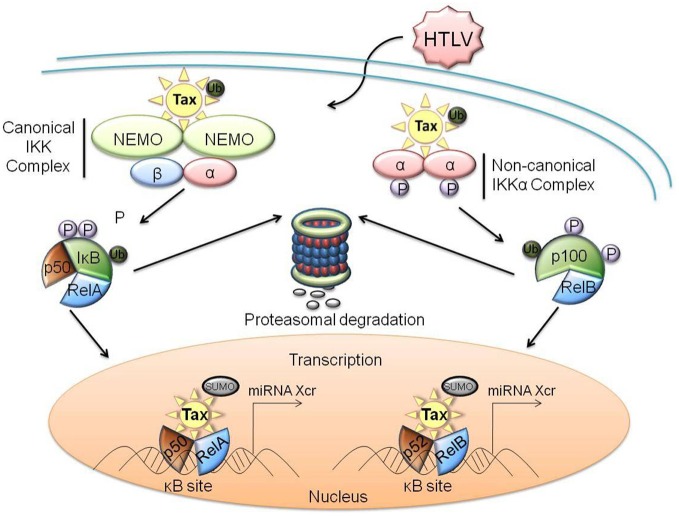
**Protein-protein interactions of HTLV-1 Tax with members of the NF-κB family of transcription factors.** Dysregulation of the canonical pathway occurs with the interaction of ubiquitinylated Tax to the cytoplasmic IKK complex, specifically binding to the IKKγ subunit. This interaction results in the phosphorylation of IκB, as well as the ubiquitination and subsequent degradation of IκB through the proteasome pathway. RelA is subsequently activated and translocates into the nucleus where SUMOylated Tax recruits RelA to Tax-nuclear bodies, driving Tax-mediated NF-κB transcription. Similarly, ubiquitinylated Tax interacts with the IKKα complex to induce the processing of p100 to p52 within the non-canonical pathway. This promotes the phosphorylation, ubiquitination, and subsequent proteasomal degradation of p100, as well as the recruitment of RelB to the nucleus for activation of Tax-mediated NF-κB transcription. Xcr, transcription.

Another mechanism by which HTLV-1 may manipulate the host cell miRNA expression profile is through the activation of host transcription factors. Important transcription factors and cellular kinases which interact directly with the viral protein Tax are CREB, serum-responsive factor (SRF), NF-κB, Cyclins D2 and D3, mitotic checkpoint regulators (MAD1), cyclin-dependent kinases (CDKs), the CDK inhibitors p16^INK4A^ and p21^(WAF1/CIP1)^, and the tumor suppressor p53 (Caron et al., 1993; Suzuki et al., 1994; Yin et al., 1995; Clemens et al., 1996; Colgin and Nyborg, 1998; Harrod et al., 1998; Yin et al., 1998; Gachon et al., 2000; Nicot et al., 2000; Xiao et al., 2001; Kashanchi and Brady, 2005; Easley et al., 2010). Of these, dramatic activation of both the canonical and non-canonical pathways of NF-κB is a hallmark of HTLV-1 infection and is a result of direct interaction between Tax and the NF-κB regulatory subunit IKKγ (Sun and Yamaoka, 2005; Yasunaga and Matsuoka, 2011). This interaction results in a constant activation of the NF-κB cascade. Furthermore, the NF-κB cascade promotes the transcription of a number of cellular genes that control a wide variety of cellular processes, including cellular proliferation and survival. The cascade is divided into two pathways, canonical and non-canonical. The two distinct IKK complexes associated with these pathways are either comprised of two subunits of IKKα, or, alternatively, one of IKKα, one of IKKβ, and two subunits of IKKγ, in the case of the non-canonical and canonical pathways, respectively. In either case, activation of the IKK complex results in the inactivation of an inhibitor of NF-κB (IκB) family protein. This in turn results in the localization of RelA and p50 (canonical pathway) or RelB and p52 (non-canonical pathway) to the nucleus. This cascade subsequently up-regulates transcription at κB promoter sites in the chromatin (Lo et al., 2006; Shembade and Harhaj, 2010). As numerous miRNA promoters are positively regulated by NF-κB, it can be inferred that the activation of NF-κB by Tax increases the expression of several host cell miRNAs (Figure 3) (Li et al., 2012b; Lukiw, 2012; Wang et al., 2012). One specific example is miR-155 which has been found to be up-regulated in HTLV-1 infected cells, as well as in a tumor necrosis factor α (TNFα) stimulated cell line through an NF-κB pathway (Bellon et al., 2009; Liu et al., 2011). While this mechanism may explain the up-regulation of several host miRNAs, examination of reporter constructs under the control of promoters from the various up-regulated miRNAs in HTLV-1 infected cells would verify exactly which of these are regulated via NF-κB.

## The role of miRNA in HTLV-1 oncogenic signaling

In addition to being the first identified human retrovirus, HTLV-1 is also currently the only known oncogenic human retrovirus causing a clinical condition known as ATL (Ruggero et al., 2010). ATL is an aggressive disease state that is highly resistant to current forms of chemotherapy and, consequently, usually results in the death of the patient (Tsukasaki et al., 2007). While the complete mechanisms of oncogenesis and tumorgenesis in HTLV-1 infected individuals has yet to be fully elucidated, a significant body of knowledge has been developed over the past 35 years. The virally encoded 40 kDa transactivator protein, Tax, has been shown to immortalize T cells both *in vitro* and *in vivo* in the absence of other viral factors and, thus, has become the focus of much research into HTLV-1-mediated oncogenesis (Pozzatti et al., 1990; Tanaka et al., 1990; Grossman et al., 1995). In recent years, however, studies have expanded beyond a Tax-centered focus and have postulated that Tax alone is not responsible for cellular transformation but rather functions in conjunction with some other viral or cellular factor. Investigations in to this arena have detailed a number of Tax interactions that may play a role in cellular transformation and of particular interest is the role of miRNA in cellular transformation (Table 1).

**Table 1:** **An abbreviated list of deregulated host miRNAs, their target mRNA, and subsequent biological effect in HTLV-1 infection**.

**Host miRNA**	**Up- or Down-regulated**	**Target mRNA**	**Biological function**
miR-21	Up	PTEN	Anti-apoptotic
miR-93	Up	p21(WAF1/CIP1)	Anti-apoptotic
		MICB	Immune Evasion
miR-132	Down	p300	Increase viral transcription
		AChE	Pro-inflammatory
miR-143-p3	Up	PKA	Proliferation
		GRα	Proliferation
miR-146a	Up	Unknown	Proliferation
miR-149	Down	p300	Increase viral transcription
miR-155	Up	TP53INP1	Anti-apoptotic
		Unknown	Increase interferon-γ
miR-873	Down	p300	Increase viral transcription

Numerous host miRNAs are either up- or down-regulated in HTLV-1 infected cells through varied molecular mechanisms. Listed here are several deregulated host miRNAs found to play roles in the oncogenic or neurodegenerative diseases associated with HTLV-1. Additionally, important cellular targeted mRNAs are listed, as well as the resultant biological effect of the miRNA deregulation for those miRNAs that have been characterized.

### Altered miRNA expression can promote oncogenesis

The current understanding of miRNA dynamics within a healthy cell indicates that the RNAi machinery functions by modulating numerous varied cellular processes simultaneously since a single miRNA can target several different mRNAs. Consequently, the up- or down-regulation of one or more miRNA will result in a corresponding shift in balance for several pathways. In the case of HTLV-1 infected cells, the virus alters the regulation of a number of miRNAs in order to promote cell survival and proliferation. While a complete understanding of viral interaction with cellular miRNAs remains to be elucidated, a number of significant interactions have been observed.

One key finding was the up-regulation of miR-93 in HTLV-1 infected cells. A number of groups have reported increased expression of miR-93 in a variety of tumors, as well as in HTLV-1 infected cell lines and ATL patient samples, indicating that this miRNA plays a significant role in cellular transformation (Yanaihara et al., 2006; Blenkiron et al., 2007; Ambs et al., 2008; Nam et al., 2008; Petrocca et al., 2008; Yeung et al., 2008; Kan et al., 2009; Li et al., 2009; Ruggero et al., 2010). In uninfected cells, miR-93 is involved in the control of Transforming Growth Factor-β (TGF-β) mediated cell cycling. MiR-93 expression is induced by the S phase regulator protein E2F1, which is then targeted and subsequently down-regulated by miR-93. Another vital cell cycle promoter, p21^(WAF1/CIP1)^, is also a target of miR-93 (Figure 1). p21^(WAF1/CIP1)^ is up-regulated by the interaction of Tax with the anaphase-promoting complex/cyclosome (APC/C) consequently causing a phenomenon known as Tax-induced rapid cellular senescence (Tax-IRS). Tax-IRS causes newly infected cells to rapidly progress through the cell cycle and ultimately undergo premature apoptosis. However, HTLV-1 transformed cells experience cell cycle arrest despite continued expression of Tax (Kuo and Giam, 2006; Yang et al., 2011a; Zhi et al., 2011). Thus, the observation that miR-93 is up-regulated in HTLV-1 cell lines and ATL samples suggests that the virus may hijack the cellular RNAi machinery in order to prevent unchecked cellular senescence and promote cellular immortalization. Furthermore, major histocompatibility complex class I chain-related B (MICB) has been identified as a significant target of miR-93 (Figure 1). MICB is a ligand recognized by the natural killer group 2 receptor member D (NKG2D), a receptor found in natural killer cells that are responsible for mediation of cell killing (Ruggero et al., 2010; Elias and Mandelboim, 2012). By inducing the over-expression of miR-93, transformed cells could effectively down-regulate MICB and, thereby, promote evasion of the host tumor surveillance activity; thus, likely contributing to the continued survival of HTLV-1 infected cells.

Yet another host miRNA, miR-223, was shown to be up-regulated in HTLV-1 infected cells from ATL patients (Bellon et al., 2009). The over-expression and increased serum levels of miR-223 has been observed in numerous *in vitro* models and clinical samples from patients with varying carcinomas (Yao et al., 2009; Li et al., 2010, 2011b, 2012a; Xu et al., 2011; Yang et al., 2011b; Kurashige et al., 2012). Moreover, miR-223 over-expression in both *in vitro* and *in vivo* gastric carcinoma models increased cell adhesion and invasion, with increased metastasis to lymph nodes (Li et al., 2011b). This increase in invasiveness and metastasis was shown to be due to miR-223 targeting of the 3′-UTR of the anti-migration factor erythrocyte membrane protein band 4.1-like 3 (EPB41L3). While this data would suggest a role of miR-223 in promoting ATL oncogenesis, other studies have found miR-223 to be an anti-oncogenic marker (Li et al., 2011a; Wu et al., 2012). One of these studies demonstrated that over-expression of miR-223 could in fact decrease cellular migration and invasiveness by targeting the neurotrophic factor Artemin (Li et al., 2011a). Furthermore, in the study that found increased expression of miR-223 from ATL patients, they also found decreased miR-223 expression in HTLV-1 infected model cell lines (Bellon et al., 2009). This contradictory data regarding miR-223 demonstrates the complicated and sometimes divergent functionalities of miRNAs due to their ability to target numerous mRNAs from varied cellular pathways. It also shows the difficulty in linking observations made from *in vitro* model cell lines to cells infected in highly complex *in vivo* systems. In order to determine the true mechanism of action with regards to miR-223 in HTLV-1 infected patients, more studies are required in the near future.

Additionally, up-regulation of miR-143-p3 has also been observed in both *in vitro* HTLV-1 infected cell lines and *in vivo* cells from ATL patients (Bellon et al., 2009). Beyond the observed up-regulation in the HTLV-1 infected cells, miR-143-p3 has also been found to be up-regulated in T-leukemic cell lines, as well as from T-leukemic cells isolated from T cell acute lymphoblastic leukemia (T-ALL) (Lv et al., 2012). From the study regarding increased miR-143-p3 in T-ALL patients, it was found that the mechanism of action for leukemogenesis by this miRNA was due to reduced cAMP activation of protein kinase A (PKA) and direct targeting of the glucocorticoid receptor α (GRα) mRNA 3′-UTR. Furthermore, the oncogenic virus, Kaposi's sarcoma associated herpesvirus (KSHV), was also found to encode a viral miRNA, miR-10Ka, that has high homology to the seed region of miR-143-p3 variants and, therefore, targets many of the same mRNAs (Gottwein et al., 2011).

The host miRNA, miR-155, has also been identified as important to HTLV-1 mediated cellular transformation. Increased expression of miR-155 in transformed cells is not surprising as it has been observed in other oncogenic viruses, such as EBV (Metzler et al., 2004). Furthermore, some viruses, specifically KSHV and Marek's disease virus, even produce viral miRNAs that mimic miR-155 (Gottwein et al., 2007; Skalsky et al., 2007; Zhao et al., 2011). Mechanistically, miR-155 targets Tumor Protein 53-Induced Nuclear Protein 1 (TP53INP1), which promotes cell cycle arrest and subsequent apoptosis (Figure 1). Therefore, the inhibition of TP53INP1 by miR-155 induces cellular transformation (Ruggero et al., 2010). Moreover, the observed up-regulation of miR-155 in viruses such as EBV is a direct result of activation of the NF-κB pathway by viral proteins. This is comparable to the mechanism of NF-κB activation via Tax described earlier.

The maximal level of NF-κB activation by Tax provides the beginning to what could prove to be another fascinating phenomenon in miRNA mediated oncogenesis as miR-146a and miR-21 are both regulated by the NF-κB pathway and are reportedly up-regulated in HTLV-1 infected cells. The up-regulation of miR-146a and miR-21 is likely important in the oncogenesis of HTLV-1 infected cells as these miRNAs have been shown to elicit anti-apoptotic functionality and are over-expressed in a number of human cancers (He et al., 2005; Volinia et al., 2006; Selcuklu et al., 2009; Tomita et al., 2012). The connection between NF-κB activation and HTLV-1 mediated regulation of cellular miRNA will require further study but could provide novel insight into the dynamics of Tax mediated cellular transformation.

### Tax down-regulates select miRNAs to promote continuous viral transcription

As described earlier, transcription of the HTLV-1 viral genome is largely carried out by the formation of the Tax/CREB/CBP/p300 complex which binds to the TRE-1. For Tax activated transcription to proceed, Tax must dimerize and recruit the CREB dimer, which interacts directly with the viral CRE in the TRE-1. Following the formation of the Tax/CREB complex, CBP/p300 is recruited in order to acetylate the histones of proximal nucleosomes and, thus, promote a chromatin remodeling event that results in an euchromatic state. This open chromatin structure provided by CBP/p300 is vital for transcription to proceed.

Recent work by Rahman et al. has provided evidence that Tax down-regulated miRNAs that target p300 (Rahman et al., 2012). Utilizing a stably integrated HTLV-1 LTR-luciferase reporter construct in Jurkat cells, they demonstrated a down-regulation of 35 different miRNAs in the presence of Tax. Amongst these, miR-149 and miR-873, which target p300, were demonstrated to be down-regulated by 7-fold and 5-fold respectively in HTLV-1 infected MT-2 cells, as compared to uninfected Jurkat cells. This observation demonstrates the role of Tax in hijacking the cellular RNAi machinery to modulate viral gene expression. The ability of Tax to manipulate the RNAi machinery is further supported by recent work from our lab, which reported that Tax significantly reduces the expression of Drosha, a crucial enzyme in the formation of mature miRNA. Through a series of transfection experiments, we demonstrated that Tax and Drosha co-localize in the nucleus and that Tax prevented the cleavage of pri-miRNA by Drosha (Van Duyne et al., 2012).

The studies reviewed here indicate that Tax-mediated regulation of miRNA expression is not simply a down-stream by-product of NF-κB or any other transcription factor activation by Tax. Rather it implicates Tax in a direct role in regulation of cellular miRNA. Even more compelling is the notion that this could provide an explanation for the low levels of Tax expression seen in older transformed cells as compared to the strong Tax expression observed in newly transformed cells. Further research into the Tax and miRNA interplay is necessary to fully elucidate the underlying molecular mechanisms; however, the data to date indicates that Tax is required to induce cellular transformation by initiating a cascade effect via miRNA dysregulation. The host cell miRNA dysregulation results in cellular immortalization and immune evasion, while still maintaining viral transcription. Furthermore, once cellular immortalization is established through the miRNA profile alteration, Tax is not required for transformation maintenance as it is difficult, if not impossible, for the cell to correct the imbalance (Jeang, 2010). The role of Tax in miRNA regulation is a recent and novel line of inquiry and continued research will be required to fully understand this exciting avenue of research.

## Altered miRNA expression can promote HAM/TSP

In addition to inducing oncogenesis, HTLV-1 up-regulated miRNAs may also contribute to the development of the other major clinical manifestation of this virus, HAM/TSP (Table 1). Specifically, miR-155, which is up-regulated in HTLV-1 infected cells, has also been associated with other neuro-inflammatory diseases such as multiple sclerosis (Paraboschi et al., 2011). This contribution of miR-155 to the onset of HAM/TSP may be in part due to its ability to increase production of interferon-γ, which is a characteristic feature of the disease (Yamano et al., 2009; Trotta et al., 2012). Furthermore, another study showed the up-regulation of miR-155 could be effectively attenuated through the treatment of the HTLV-1 infected MT-2 cell line with a high dose ascorbic acid regimen. Moreover, treatment with high doses of ascorbic acid also exhibited significant cell death and anti-proliferative effects on both *in vitro* and *ex vivo* cultures of HTLV-1 infected cells (Moens et al., 2012).

Another miRNA that is likely involved in the HAM/TSP pathogenesis is miR-132. Specifically, miR-132 was shown to be down-regulated in HTLV-1 infected cell lines, samples from ATL patients, and Tax transfected cell lines (Bellon et al., 2009; Rahman et al., 2012). Functionally, miR-132 has been shown to target acetylcholine esterase (AChE) mRNA, and therefore, the down-regulation of miR-132 boosts AChE activity and subsequently reduces acetylcholine levels (Shaked et al., 2009). It has been shown that in response to pro-inflammatory stimulus, the vagus nerve will release acetylcholine which in turn will attenuate pro-inflammatory cytokine release such as TNF, IL-6, and IL-18 from macrophages and can limit the influx of activated T cells into simple motor neuron lesions (Borovikova et al., 2000; Nicolussi et al., 2009). The activation of the anti-inflammatory pathway by acetylcholine has been demonstrated to be via binding to the nicotinic acetylcholine receptor α-7 subunit (Wang et al., 2003). Moreover, the down-regulation of miR-132 has also been observed in tissue samples of Alzheimer's disease patients linking this miRNA to another pro-inflammatory neurodegenerative condition (Cogswell et al., 2008). It is also interesting to note that miR-132 targets p300, which as previously described is essential to HTLV-1 proviral transcription and, therefore, likely boosts viral replication. Again, this potential duel functionality of miR-132 demonstrates the complexity of the varied potential effects-induced by dysregulation host miRNA profiles.

## Conclusions

The manipulation of host miRNAs and RNAi machinery, as well as encoding of viral miRNAs by human pathogenic viruses has only recently come to light. Due to the diverse cellular pathways controlled by miRNAs, the hijacking of the RNAi molecular machinery and alteration of host miRNA profiles represents a significant and diverse mechanism by which viruses can progress through their life cycle and effect varied disease states in the host organism. Here, we examined recent findings that show the human retrovirus, HTLV-1, does indeed alter the miRNA profiles of infected cells which contributes to cellular transformation and leads to the associated ATL and HAM/TSP disease states. Furthermore, we show that alteration of chromatin by viral proteins and host cell miRNAs can contribute to the dysregulation host cell miRNA expression and likely serves as a key mechanism by which the virus manipulates host miRNA profiles. While recent discoveries validate the importance of HTLV-1 mediated variation in miRNA levels, there is still much to be elucidated in this novel field of study. Moreover, a better understanding of the molecular mechanisms by which this viral manipulation occurs will aid in the identification of potential points of therapeutic intervention that could help eradicate this pathogen from infected individuals.

### Conflict of interest statement

The authors declare that the research was conducted in the absence of any commercial or financial relationships that could be construed as a potential conflict of interest.

## References

[B1] Ahlenstiel C. L. Lim H. G. Cooper D. A. Ishida T. Kelleher A. D. Suzuki K. (2012). Direct evidence of nuclear Argonaute distribution during transcriptional silencing links the actin cytoskeleton to nuclear RNAi machinery in human cells. Nucleic Acids Res. 40, 1579–1595 10.1093/nar/gkr891 22064859 PMC3287199

[B2] Althaus C. F. Vongrad V. Niederost B. Joos B. Di Giallonardo F. Rieder P. (2012). Tailored enrichment strategy detects low abundant small noncoding RNAs in HIV-1 infected cells. Retrovirology 9:27 10.1186/1742-4690-9-27 22458358 PMC3341194

[B3] Ambs S. Prueitt R. L. Yi M. Hudson R. S. Howe T. M. Petrocca F. (2008). Genomic profiling of microRNA and messenger RNA reveals deregulated microRNA expression in prostate cancer. Cancer Res. 68, 6162–6170 10.1158/0008-5472.CAN-08-0144 18676839 PMC2597340

[B4] Bansal A. Carlson J. Yan J. Akinsiku O. T. Schaefer M. Sabbaj S. (2010). CD8 T cell response and evolutionary pressure to HIV-1 cryptic epitopes derived from antisense transcription. J. Exp. Med. 207, 51–59 10.1084/jem.20092060 20065064 PMC2812545

[B5] Baranger A. M. Palmer C. R. Hamm M. K. Giebler H. A. Brauweiler A. Nyborg J. K. (1995). Mechanism of DNA-binding enhancement by the human T-cell leukaemia virus transactivator Tax. Nature 376, 606–608 10.1038/376606a0 7637812

[B6] Barth S. Pfuhl T. Mamiani A. Ehses C. Roemer K. Kremmer E. (2008). Epstein-Barr virus-encoded microRNA miR-BART2 down-regulates the viral DNA polymerase BALF5. Nucleic Acids Res. 36, 666–675 10.1093/nar/gkm1080 18073197 PMC2241876

[B7] Bellon M. Lepelletier Y. Hermine O. Nicot C. (2009). Deregulation of microRNA involved in hematopoiesis and the immune response in HTLV-I adult T-cell leukemia. Blood 113, 4914–4917 10.1182/blood-2008-11-189845 19246560 PMC2686141

[B8] Bennasser Y. Le S. Y. Benkirane M. Jeang K. T. (2005). Evidence that HIV-1 encodes an siRNA and a suppressor of RNA silencing. Immunity 22, 607–619 10.1016/j.immuni.2005.03.010 15894278

[B9] Bentley K. Deacon N. Sonza S. Zeichner S. Churchill M. (2004). Mutational analysis of the HIV-1 LTR as a promoter of negative sense transcription. Arch. Virol. 149, 2277–2294 10.1007/s00705-004-0386-8 15338321

[B10] Blenkiron C. Goldstein L. D. Thorne N. P. Spiteri I. Chin S. F. Dunning M. J. (2007). MicroRNA expression profiling of human breast cancer identifies new markers of tumor subtype. Genome Biol. 8:R214 10.1186/gb-2007-8-10-r214 17922911 PMC2246288

[B11] Bogenberger J. M. Laybourn P. J. (2008). Human T Lymphotropic Virus Type 1 protein Tax reduces histone levels. Retrovirology 5:9 10.1186/1742-4690-5-9 18237376 PMC2276518

[B12] Bogerd H. P. Karnowski H. W. Cai X. Shin J. Pohlers M. Cullen B. R. (2010). A mammalian herpesvirus uses noncanonical expression and processing mechanisms to generate viral MicroRNAs. Mol. Cell 37, 135–142 10.1016/j.molcel.2009.12.016 20129062 PMC2818755

[B13] Borovikova L. V. Ivanova S. Zhang M. Yang H. Botchkina G. I. Watkins L. R. (2000). Vagus nerve stimulation attenuates the systemic inflammatory response to endotoxin. Nature 405, 458–462 10.1038/35013070 10839541

[B14] Botos I. Liu L. Wang Y. Segal D. M. Davies D. R. (2009). The toll-like receptor 3:dsRNA signaling complex. Biochim. Biophys. Acta 1789, 667–674 10.1016/j.bbagrm.2009.06.005 19595807 PMC2784288

[B15] Briquet S. Vaquero C. (2002). Immunolocalization studies of an antisense protein in HIV-1-infected cells and viral particles. Virology 292, 177–184 10.1006/viro.2001.1224 11878921

[B16] Buhler M. Moazed D. (2007). Transcription and RNAi in heterochromatic gene silencing. Nat. Struct. Mol. Biol. 14, 1041–1048 10.1038/nsmb1315 17984966

[B17] Cai X. Hagedorn C. H. Cullen B. R. (2004). Human microRNAs are processed from capped, polyadenylated transcripts that can also function as mRNAs. RNA 10, 1957–1966 10.1261/rna.7135204 15525708 PMC1370684

[B18] Cameron J. E. Fewell C. Yin Q. McBride J. Wang X. Lin Z. (2008). Epstein-Barr virus growth/latency III program alters cellular microRNA expression. Virology 382, 257–266 10.1016/j.virol.2008.09.018 18950829 PMC2640950

[B19] Carissimi C. Fulci V. Macino G. (2009). MicroRNAs: novel regulators of immunity. Autoimmun. Rev. 8, 520–524 10.1016/j.autrev.2009.01.008 19200459

[B20] Caron C. Rousset R. Beraud C. Moncollin V. Egly J. M. Jalinot P. (1993). Functional and biochemical interaction of the HTLV-I Tax1 transactivator with TBP. EMBO J. 12, 4269–4278 8223437 10.1002/j.1460-2075.1993.tb06111.x PMC413723

[B21] Castanotto D. Tommasi S. Li M. Li H. Yanow S. Pfeifer G. P. (2005). Short hairpin RNA-directed cytosine (CpG) methylation of the RASSF1A gene promoter in HeLa cells. Mol. Ther. 12, 179–183 10.1016/j.ymthe.2005.03.003 15963934

[B22] Cedar H. Bergman Y. (2009). Linking DNA methylation and histone modification: patterns and paradigms. Nat. Rev. Genet. 10, 295–304 10.1038/nrg2540 19308066

[B23] Chen C. Z. Li L. Lodish H. F. Bartel D. P. (2004). MicroRNAs modulate hematopoietic lineage differentiation. Science 303, 83–86 10.1126/science.1091903 14657504

[B24] Chendrimada T. P. Gregory R. I. Kumaraswamy E. Norman J. Cooch N. Nishikura K. (2005). TRBP recruits the Dicer complex to Ago2 for microRNA processing and gene silencing. Nature 436, 740–744 10.1038/nature03868 15973356 PMC2944926

[B25] Chiba H. Muramatsu M. Nomoto A. Kato H. (1994). Two human homologues of Saccharomyces cerevisiae SWI2/SNF2 and *Drosophila brahma* are transcriptional coactivators cooperating with the estrogen receptor and the retinoic acid receptor. Nucleic Acids Res. 22, 1815–1820 10.1093/nar/22.10.1815 8208605 PMC308079

[B27] Chu C. Y. Rana T. M. (2006). Translation repression in human cells by microRNA-induced gene silencing requires RCK/p54. PLoS Biol. 4:e210 10.1371/journal.pbio.0040210 16756390 PMC1475773

[B26] Chua J. H. Armugam A. Jeyaseelan K. (2009). MicroRNAs: biogenesis, function and applications. Curr. Opin. Mol. Ther. 11, 189–199 19330724

[B28] Clemens K. E. Piras G. Radonovich M. F. Choi K. S. Duvall J. F. Dejong J. (1996). Interaction of the human T-cell lymphotropic virus type 1 tax transactivator with transcription factor IIA. Mol. Cell. Biol. 16, 4656–4664 8756622 10.1128/mcb.16.9.4656 PMC231465

[B29] Clerc I. Laverdure S. Torresilla C. Landry S. Borel S. Vargas A. (2011). Polarized expression of the membrane ASP protein derived from HIV-1 antisense transcription in T cells. Retrovirology 8:74 10.1186/1742-4690-8-74 21929758 PMC3182985

[B30] Cobb B. S. Hertweck A. Smith J. O'Connor E. Graf D. Cook T. (2006). A role for Dicer in immune regulation. J. Exp. Med. 203, 2519–2527 10.1084/jem.20061692 17060477 PMC2118134

[B31] Cogswell J. P. Ward J. Taylor I. A. Waters M. Shi Y. Cannon B. (2008). Identification of miRNA changes in Alzheimer's disease brain and CSF yields putative biomarkers and insights into disease pathways. J. Alzheimers Dis. 14, 27–41 18525125 10.3233/jad-2008-14103

[B32] Colgin M. A. Nyborg J. K. (1998). The human T-cell leukemia virus type 1 oncoprotein Tax inhibits the transcriptional activity of c-Myb through competition for the CREB binding protein. J. Virol. 72, 9396–9399 9765496 10.1128/jvi.72.11.9396-9399.1998 PMC110368

[B33] Curtale G. Citarella F. Carissimi C. Goldoni M. Carucci N. Fulci V. (2010). An emerging player in the adaptive immune response: microRNA-146a is a modulator of IL-2 expression and activation-induced cell death in T lymphocytes. Blood 115, 265–273 10.1182/blood-2009-06-225987 19965651

[B34] De La Serna I. L. Ohkawa Y. Imbalzano A. N. (2006). Chromatin remodelling in mammalian differentiation: lessons from ATP-dependent remodellers. Nat. Rev. Genet. 7, 461–473 10.1038/nrg1882 16708073

[B35] Denli A. M. Tops B. B. Plasterk R. H. Ketting R. F. Hannon G. J. (2004). Processing of primary microRNAs by the Microprocessor complex. Nature 432, 231–235 10.1038/nature03049 15531879

[B36] Easley R. Carpio L. Guendel I. Klase Z. Choi S. Kehn-Hall K. (2010). Human T-lymphotropic virus type 1 transcription and chromatin-remodeling complexes. J. Virol. 84, 4755–4768 10.1128/JVI.00851-09 20164218 PMC2863730

[B37] Easow G. Teleman A. A. Cohen S. M. (2007). Isolation of microRNA targets by miRNP immunopurification. RNA 13, 1198–1204 10.1261/rna.563707 17592038 PMC1924889

[B38] Elias S. Mandelboim O. (2012). Battle of the midgets: innate microRNA networking. RNA Biol. 9, 792–798 10.4161/rna.19717 22617882

[B39] Faller M. Guo F. (2008). MicroRNA biogenesis: there's more than one way to skin a cat. Biochim. Biophys. Acta 1779, 663–667 10.1016/j.bbagrm.2008.08.005 18778799 PMC2633599

[B40] Fan S. Zhang M. Q. Zhang X. (2008). Histone methylation marks play important roles in predicting the methylation status of CpG islands. Biochem. Biophys. Res. Commun. 374, 559–564 10.1016/j.bbrc.2008.07.077 18656446 PMC2974564

[B41] Faraoni I. Antonetti F. R. Cardone J. Bonmassar E. (2009). miR-155 gene: a typical multifunctional microRNA. Biochim. Biophys. Acta 1792, 497–505 10.1016/j.bbadis.2009.02.013 19268705

[B42] Fazi F. Rosa A. Fatica A. Gelmetti V. De Marchis M. L. Nervi C. (2005). A minicircuitry comprised of microRNA-223 and transcription factors NFI-A and C/EBPalpha regulates human granulopoiesis. Cell 123, 819–831 10.1016/j.cell.2005.09.023 16325577

[B43] Gachon F. Thebault S. Peleraux A. Devaux C. Mesnard J. M. (2000). Molecular interactions involved in the transactivation of the human T-cell leukemia virus type 1 promoter mediated by Tax and CREB-2 (ATF-4). Mol. Cell. Biol. 20, 3470–3481 10.1128/MCB.20.10.3470-3481.2000 10779337 PMC85640

[B44] Gallo R. C. (2011). Research and discovery of the first human cancer virus, HTLV-1. Best Pract. Res. Clin. Haematol. 24, 559–565 10.1016/j.beha.2011.09.012 22127321

[B45] Gatto G. Rossi A. Rossi D. Kroening S. Bonatti S. Mallardo M. (2008). Epstein-Barr virus latent membrane protein 1 trans-activates miR-155 transcription through the NF-kappaB pathway. Nucleic Acids Res. 36, 6608–6619 10.1093/nar/gkn666 18940871 PMC2582607

[B46] Giebler H. A. Loring J. E. Van Orden K. Colgin M. A. Garrus J. E. Escudero K. W. (1997). Anchoring of CREB binding protein to the human T-cell leukemia virus type 1 promoter: a molecular mechanism of Tax transactivation. Mol. Cell. Biol. 17, 5156–5164 9271393 10.1128/mcb.17.9.5156 PMC232366

[B47] Gottwein E. Corcoran D. L. Mukherjee N. Skalsky R. L. Hafner M. Nusbaum J. D. (2011). Viral microRNA targetome of KSHV-infected primary effusion lymphoma cell lines. Cell Host Microbe 10, 515–526 10.1016/j.chom.2011.09.012 22100165 PMC3222872

[B48] Gottwein E. Mukherjee N. Sachse C. Frenzel C. Majoros W. H. Chi J. T. (2007). A viral microRNA functions as an orthologue of cellular miR-155. Nature 450, 1096–1099 10.1038/nature05992 18075594 PMC2614920

[B49] Griffiths-Jones S. Grocock R. J. Van Dongen S. Bateman A. Enright A. J. (2006). miRBase: microRNA sequences, targets and gene nomenclature. Nucleic Acids Res. 34, D140–D144 10.1093/nar/gkj112 16381832 PMC1347474

[B50] Griffiths-Jones S. Saini H. K. Van Dongen S. Enright A. J. (2008). miRBase: tools for microRNA genomics. Nucleic Acids Res. 36, D154–D158 10.1093/nar/gkm952 17991681 PMC2238936

[B51] Grossman W. J. Kimata J. T. Wong F. H. Zutter M. Ley T. J. Ratner L. (1995). Development of leukemia in mice transgenic for the tax gene of human T-cell leukemia virus type I. Proc. Natl. Acad. Sci. U.S.A. 92, 1057–1061 7862633 10.1073/pnas.92.4.1057 PMC42636

[B52] Guo H. Ingolia N. T. Weissman J. S. Bartel D. P. (2010). Mammalian microRNAs predominantly act to decrease target mRNA levels. Nature 466, 835–840 10.1038/nature09267 20703300 PMC2990499

[B53] Haase A. D. Jaskiewicz L. Zhang H. Laine S. Sack R. Gatignol A. (2005). TRBP, a regulator of cellular PKR and HIV-1 virus expression, interacts with Dicer and functions in RNA silencing. EMBO Rep. 6, 961–967 10.1038/sj.embor.7400509 16142218 PMC1369185

[B54] Hafner M. Landthaler M. Burger L. Khorshid M. Hausser J. Berninger P. (2010). Transcriptome-wide identification of RNA-binding protein and microRNA target sites by PAR-CLIP. Cell 141, 129–141 10.1016/j.cell.2010.03.009 20371350 PMC2861495

[B55] Hakim S. T. Alsayari M. Mclean D. C. Saleem S. Addanki K. C. Aggarwal M. (2008). A large number of the human microRNAs target lentiviruses, retroviruses, and endogenous retroviruses. Biochem. Biophys. Res. Commun. 369, 357–362 10.1016/j.bbrc.2008.02.025 18282469

[B56] Han J. Lee Y. Yeom K. H. Kim Y. K. Jin H. Kim V. N. (2004). The Drosha-DGCR8 complex in primary microRNA processing. Genes Dev. 18, 3016–3027 10.1101/gad.1262504 15574589 PMC535913

[B57] Harrod R. Tang Y. Nicot C. Lu H. S. Vassilev A. Nakatani Y. (1998). An exposed KID-like domain in human T-cell lymphotropic virus type 1 Tax is responsible for the recruitment of coactivators CBP/p300. Mol. Cell. Biol. 18, 5052–5061 9710589 10.1128/mcb.18.9.5052 PMC109090

[B58] He H. Jazdzewski K. Li W. Liyanarachchi S. Nagy R. Volinia S. (2005). The role of microRNA genes in papillary thyroid carcinoma. Proc. Natl. Acad. Sci. U.S.A. 102, 19075–19080 10.1073/pnas.0509603102 16365291 PMC1323209

[B59] Hemelaar J. Bex F. Booth B. Cerundolo V. Mcmichael A. Daenke S. (2001). Human T-cell leukemia virus type 1 Tax protein binds to assembled nuclear proteasomes and enhances their proteolytic activity. J. Virol. 75, 11106–11115 10.1128/JVI.75.22.11106-11115.2001 11602750 PMC114690

[B60] Houzet L. Yeung M. L. De Lame V. Desai D. Smith S. M. Jeang K. T. (2008). MicroRNA profile changes in human immunodeficiency virus type 1 (HIV-1) seropositive individuals. Retrovirology 5:118 10.1186/1742-4690-5-118 19114009 PMC2644721

[B61] Huang B. Zhao J. Lei Z. Shen S. Li D. Shen G. X. (2009). miR-142-3p restricts cAMP production in CD4+CD25- T cells and CD4+CD25+ TREG cells by targeting AC9 mRNA. EMBO Rep. 10, 180–185 10.1038/embor.2008.224 19098714 PMC2637310

[B62] Huang J. Wang F. Argyris E. Chen K. Liang Z. Tian H. (2007). Cellular microRNAs contribute to HIV-1 latency in resting primary CD4+ T lymphocytes. Nat. Med. 13, 1241–1247 10.1038/nm1639 17906637

[B63] Ivanovska I. Ball A. S. Diaz R. L. Magnus J. F. Kibukawa M. Schelter J. M. (2008). MicroRNAs in the miR-106b family regulate p21/CDKN1A and promote cell cycle progression. Mol. Cell. Biol. 28, 2167–2174 10.1128/MCB.01977-07 18212054 PMC2268421

[B64] Janowski B. A. Huffman K. E. Schwartz J. C. Ram R. Nordsell R. Shames D. S. (2006). Involvement of AGO1 and AGO2 in mammalian transcriptional silencing. Nat. Struct. Mol. Biol. 13, 787–792 10.1038/nsmb1140 16936728

[B65] Jeang K. T. (2010). Human T cell leukemia virus type 1 (HTLV-1) and oncogene or oncomiR addiction? Oncotarget 1, 453–456 21311101 10.18632/oncotarget.179 PMC3058865

[B66] Jeang K. T. Boros I. Brady J. Radonovich M. Khoury G. (1988). Characterization of cellular factors that interact with the human T-cell leukemia virus type I p40x-responsive 21-base-pair sequence. J. Virol. 62, 4499–4509 3263510 10.1128/jvi.62.12.4499-4509.1988 PMC253560

[B67] Johnnidis J. B. Harris M. H. Wheeler R. T. Stehling-Sun S. Lam M. H. Kirak O. (2008). Regulation of progenitor cell proliferation and granulocyte function by microRNA-223. Nature 451, 1125–1129 10.1038/nature06607 18278031

[B68] Kamoi K. Yamamoto K. Misawa A. Miyake A. Ishida T. Tanaka Y. (2006). SUV39H1 interacts with HTLV-1 Tax and abrogates Tax transactivation of HTLV-1 LTR. Retrovirology 3:5 10.1186/1742-4690-3-5 16409643 PMC1363732

[B69] Kan T. Sato F. Ito T. Matsumura N. David S. Cheng Y. (2009). The miR-106b-25 polycistron, activated by genomic amplification, functions as an oncogene by suppressing p21 and Bim. Gastroenterology 136, 1689–1700 19422085 10.1053/j.gastro.2009.02.002 PMC2887605

[B70] Kaplan J. E. Osame M. Kubota H. Igata A. Nishitani H. Maeda Y. (1990). The risk of development of HTLV-I-associated myelopathy/tropical spastic paraparesis among persons infected with HTLV-I. J. Acquir. Immune. Defic. Syndr. 3, 1096–1101 2213510

[B71] Kashanchi F. Brady J. N. (2005). Transcriptional and post-transcriptional gene regulation of HTLV-1. Oncogene 24, 5938–5951 10.1038/sj.onc.1208973 16155601

[B72] Kashanchi F. Duvall J. F. Kwok R. P. Lundblad J. R. Goodman R. H. Brady J. N. (1998). The coactivator CBP stimulates human T-cell lymphotrophic virus type I Tax transactivation *in vitro*. J. Biol. Chem. 273, 34646–34652 10.1074/jbc.273.51.34646 9852138

[B73] Kato H. Goto D. B. Martienssen R. A. Urano T. Furukawa K. Murakami Y. (2005). RNA polymerase II is required for RNAi-dependent heterochromatin assembly. Science 309, 467–469 10.1126/science.1114955 15947136

[B74] Kawamata T. Tomari Y. (2010). Making RISC. Trends Biochem. Sci. 35, 368–376 10.1016/j.tibs.2010.03.009 20395147

[B75] Kehn K. Fuente Cde L. Strouss K. Berro R. Jiang H. Brady J. (2005). The HTLV-I Tax oncoprotein targets the retinoblastoma protein for proteasomal degradation. Oncogene 24, 525–540 10.1038/sj.onc.1208105 15580311

[B76] Kfoury Y. Nasr R. Journo C. Mahieux R. Pique C. Bazarbachi A. (2012). The multifaceted oncoprotein Tax: subcellular localization, posttranslational modifications, and NF-kappaB activation. Adv. Cancer Res. 113, 85–120 10.1016/B978-0-12-394280-7.00003-8 22429853

[B77] Kim D. H. Villeneuve L. M. Morris K. V. Rossi J. J. (2006). Argonaute-1 directs siRNA-mediated transcriptional gene silencing in human cells. Nat. Struct. Mol. Biol. 13, 793–797 10.1038/nsmb1142 16936726

[B78] Kim V. N. (2004). MicroRNA precursors in motion: exportin-5 mediates their nuclear export. Trends Cell Biol. 14, 156–159 15134074 10.1016/j.tcb.2004.02.006

[B79] Kincaid R. P. Burke J. M. Sullivan C. S. (2012). RNA virus microRNA that mimics a B-cell oncomiR. Proc. Natl. Acad. Sci. U.S.A. 109, 3077–3082 10.1073/pnas.1116107109 22308400 PMC3286953

[B80] Klase Z. Kale P. Winograd R. Gupta M. V. Heydarian M. Berro R. (2007). HIV-1 TAR element is processed by Dicer to yield a viral micro-RNA involved in chromatin remodeling of the viral LTR. BMC Mol. Biol. 8:63 10.1186/1471-2199-8-63 17663774 PMC1955452

[B81] Klase Z. Winograd R. Davis J. Carpio L. Hildreth R. Heydarian M. (2009). HIV-1 TAR miRNA protects against apoptosis by altering cellular gene expression. Retrovirology 6:18 10.1186/1742-4690-6-18 19220914 PMC2654423

[B82] Kobayashi-Ishihara M. Yamagishi M. Hara T. Matsuda Y. Takahashi R. Miyake A. (2012). HIV-1-encoded antisense RNA suppresses viral replication for a prolonged period. Retrovirology 9:38 10.1186/1742-4690-9-38 22569184 PMC3410806

[B83] Kok K. H. Lei T. Jin D. Y. (2010). Identification and validation of the cellular targets of virus-encoded microRNAs. Methods Mol. Biol. 667, 319–326 10.1007/978-1-60761-811-9_21 20827543

[B84] Kozomara A. Griffiths-Jones S. (2011). miRBase: integrating microRNA annotation and deep-sequencing data. Nucleic Acids Res. 39, D152–D157 10.1093/nar/gkq1027 21037258 PMC3013655

[B85] Kuo Y. L. Giam C. Z. (2006). Activation of the anaphase promoting complex by HTLV-1 tax leads to senescence. EMBO J. 25, 1741–1752 10.1038/sj.emboj.7601054 16601696 PMC1440834

[B86] Kurashige J. Watanabe M. Iwatsuki M. Kinoshita K. Saito S. Hiyoshi Y. (2012). Overexpression of microRNA-223 regulates the ubiquitin ligase FBXW7 in oesophageal squamous cell carcinoma. Br. J. Cancer 106, 182–188 10.1038/bjc.2011.509 22108521 PMC3251856

[B87] Kwok R. P. Laurance M. E. Lundblad J. R. Goldman P. S. Shih H. Connor L. M. (1996). Control of cAMP-regulated enhancers by the viral transactivator Tax through CREB and the co-activator CBP. Nature 380, 642–646 10.1038/380642a0 8602268

[B88] Lal A. Navarro F. Maher C. A. Maliszewski L. E. Yan N. O'Day E. (2009). miR-24 Inhibits cell proliferation by targeting E2F2, MYC, and other cell-cycle genes via binding to “seedless” 3' UTR microRNA recognition elements. Mol. Cell 35, 610–625 10.1016/j.molcel.2009.08.020 19748357 PMC2757794

[B89] Landry S. Halin M. Lefort S. Audet B. Vaquero C. Mesnard J. M. (2007). Detection, characterization and regulation of antisense transcripts in HIV-1. Retrovirology 4:71 10.1186/1742-4690-4-71 17910760 PMC2099442

[B90] Larocca D. Chao L. A. Seto M. H. Brunck T. K. (1989). Human T-cell leukemia virus minus strand transcription in infected T-cells. Biochem. Biophys. Res. Commun. 163, 1006–1013 10.1016/0006-291X(89)92322-X 2476979

[B91] Lecellier C. H. Dunoyer P. Arar K. Lehmann-Che J. Eyquem S. Himber C. (2005). A cellular microRNA mediates antiviral defense in human cells. Science 308, 557–560 10.1126/science.1108784 15845854

[B92] Lee Y. Ahn C. Han J. Choi H. Kim J. Yim J. (2003). The nuclear RNase III Drosha initiates microRNA processing. Nature 425, 415–419 10.1038/nature01957 14508493

[B93] Lefebvre G. Desfarges S. Uyttebroeck F. Munoz M. Beerenwinkel N. Rougemont J. (2011). Analysis of HIV-1 expression level and sense of transcription by high-throughput sequencing of the infected cell. J. Virol. 85, 6205–6211 10.1128/JVI.00252-11 21507965 PMC3126515

[B94] Leung A. K. Calabrese J. M. Sharp P. A. (2006). Quantitative analysis of Argonaute protein reveals microRNA-dependent localization to stress granules. Proc. Natl. Acad. Sci. U.S.A. 103, 18125–18130 10.1073/pnas.0608845103 17116888 PMC1838717

[B95] Li B. S. Zhao Y. L. Guo G. Li W. Zhu E. D. Luo X. (2012a). Plasma microRNAs, miR-223, miR-21 and miR-218, as novel potential biomarkers for gastric cancer detection. PLoS ONE 7:e41629 10.1371/journal.pone.0041629 22860003 PMC3408505

[B96] Li J. Wang K. Chen X. Meng H. Song M. Wang Y. (2012b). Transcriptional activation of microRNA-34a by NF-kappa B in human esophageal cancer cells. BMC Mol. Biol. 13:4 10.1186/1471-2199-13-4 22292433 PMC3311059

[B97] Li Q. J. Chau J. Ebert P. J. Sylvester G. Min H. Liu G. (2007). miR-181a is an intrinsic modulator of T cell sensitivity and selection. Cell 129, 147–161 10.1016/j.cell.2007.03.008 17382377

[B98] Li S. Li Z. Guo F. Qin X. Liu B. Lei Z. (2011a). miR-223 regulates migration and invasion by targeting Artemin in human esophageal carcinoma. J. Biomed. Sci. 18:24 10.1186/1423-0127-18-24 21453483 PMC3080798

[B99] Li X. Zhang Y. Zhang H. Liu X. Gong T. Li M. (2011b). miRNA-223 promotes gastric cancer invasion and metastasis by targeting tumor suppressor EPB41L3. Mol. Cancer Res. 9, 824–833 10.1158/1541-7786.MCR-10-0529 21628394

[B100] Li X. Zhang Y. Ding J. Wu K. Fan D. (2010). Survival prediction of gastric cancer by a seven-microRNA signature. Gut 59, 579–585 10.1136/gut.2008.175497 19951901

[B101] Li Y. Tan W. Neo T. W. Aung M. O. Wasser S. Lim S. G. (2009). Role of the miR-106b-25 microRNA cluster in hepatocellular carcinoma. Cancer Sci. 100, 1234–1242 10.1111/j.1349-7006.2009.01164.x 19486339

[B102] Lin J. Cullen B. R. (2007). Analysis of the interaction of primate retroviruses with the human RNA interference machinery. J. Virol. 81, 12218–12226 10.1128/JVI.01390-07 17855543 PMC2169020

[B103] Liu J. Valencia-Sanchez M. A. Hannon G. J. Parker R. (2005). MicroRNA-dependent localization of targeted mRNAs to mammalian P-bodies. Nat. Cell Biol. 7, 719–723 10.1038/ncb1274 15937477 PMC1855297

[B104] Liu S. Yang Y. Wu J. (2011). TNFalpha-induced up-regulation of miR-155 inhibits adipogenesis by down-regulating early adipogenic transcription factors. Biochem. Biophys. Res. Commun. 414, 618–624 10.1016/j.bbrc.2011.09.131 21986534

[B105] Lo J. C. Basak S. James E. S. Quiambo R. S. Kinsella M. C. Alegre M. L. (2006). Coordination between NF-kappaB family members p50 and p52 is essential for mediating LTbetaR signals in the development and organization of secondary lymphoid tissues. Blood 107, 1048–1055 10.1182/blood-2005-06-2452 16195333 PMC1895903

[B106] Loffler D. Brocke-Heidrich K. Pfeifer G. Stocsits C. Hackermuller J. Kretzschmar A. K. (2007). Interleukin-6 dependent survival of multiple myeloma cells involves the Stat3-mediated induction of microRNA-21 through a highly conserved enhancer. Blood 110, 1330–1333 10.1182/blood-2007-03-081133 17496199

[B107] Lu F. Weidmer A. Liu C. G. Volinia S. Croce C. M. Lieberman P. M. (2008). Epstein-Barr virus-induced miR-155 attenuates NF-kappaB signaling and stabilizes latent virus persistence. J. Virol. 82, 10436–10443 10.1128/JVI.00752-08 18753206 PMC2573162

[B108] Ludwig L. B. Ambrus J. L. Jr. Krawczyk K. A. Sharma S. Brooks S. Hsiao C. B. (2006). Human Immunodeficiency Virus-Type 1 LTR DNA contains an intrinsic gene producing antisense RNA and protein products. Retrovirology 3:80 10.1186/1742-4690-3-80 17090330 PMC1654176

[B109] Lukiw W. J. (2012). NF-small ka, CyrillicB-regulated micro RNAs (miRNAs) in primary human brain cells. Exp. Neurol. 235, 484–490 10.1016/j.expneurol.2011.11.022 22138609 PMC3321120

[B110] Lv M. Zhang X. Jia H. Li D. Zhang B. Zhang H. (2012). An oncogenic role of miR-142-3p in human T-cell acute lymphoblastic leukemia (T-ALL) by targeting glucocorticoid receptor-alpha and cAMP/PKA pathways. Leukemia 26, 769–777 10.1038/leu.2011.273 21979877

[B111] Lytle J. R. Yario T. A. Steitz J. A. (2007). Target mRNAs are repressed as efficiently by microRNA-binding sites in the 5' UTR as in the 3' UTR. Proc. Natl. Acad. Sci. U.S.A. 104, 9667–9672 10.1073/pnas.0703820104 17535905 PMC1887587

[B112] Macrae I. J. Li F. Zhou K. Cande W. Z. Doudna J. A. (2006). Structure of Dicer and mechanistic implications for RNAi. Cold Spring Harb. Symp. Quant. Biol. 71, 73–80 10.1101/sqb.2006.71.042 17381283

[B113] Mahieux R. Gessain A. (2007). Adult T-cell leukemia/lymphoma and HTLV-1. Curr. Hematol. Malig. Rep. 2, 257–264 10.1007/s11899-007-0035-x 20425378

[B114] Mani K. S. Mani A. J. Montgomery R. D. (1969). A spastic paraplegic syndrome in South India. J. Neurol. Sci. 9, 179–199 4185984 10.1016/0022-510x(69)90067-7

[B115] Meissner A. Mikkelsen T. S. Gu H. Wernig M. Hanna J. Sivachenko A. (2008). Genome-scale DNA methylation maps of pluripotent and differentiated cells. Nature 454, 766–770 10.1038/nature07107 18600261 PMC2896277

[B116] Merkenschlager M. Wilson C. B. (2008). RNAi and chromatin in T cell development and function. Curr. Opin. Immunol. 20, 131–138 10.1016/j.coi.2008.03.013 18440793

[B117] Merkerova M. Belickova M. Bruchova H. (2008). Differential expression of microRNAs in hematopoietic cell lineages. Eur. J. Haematol. 81, 304–310 10.1111/j.1600-0609.2008.01111.x 18573170

[B118] Mette M. F. Aufsatz W. Van Der Winden J. Matzke M. A. Matzke A. J. (2000). Transcriptional silencing and promoter methylation triggered by double-stranded RNA. EMBO J. 19, 5194–5201 10.1093/emboj/19.19.5194 11013221 PMC302106

[B119] Metzler M. Wilda M. Busch K. Viehmann S. Borkhardt A. (2004). High expression of precursor microRNA-155/BIC RNA in children with Burkitt lymphoma. Genes Chromosomes Cancer 39, 167–169 10.1002/gcc.10316 14695998

[B120] Michael N. L. Vahey M. T. D'arcy L. Ehrenberg P. K. Mosca J. D. Rappaport J. (1994). Negative-strand RNA transcripts are produced in human immunodeficiency virus type 1-infected cells and patients by a novel promoter downregulated by Tat. J. Virol. 68, 979–987 8289399 10.1128/jvi.68.2.979-987.1994 PMC236536

[B121] Miller R. H. (1988). Human immunodeficiency virus may encode a novel protein on the genomic DNA plus strand. Science 239, 1420–1422 10.1126/science.3347840 3347840

[B122] Moens B. Decanine D. Menezes S. M. Khouri R. Silva-Santos G. Lopez G. (2012). Ascorbic acid has superior *ex vivo* antiproliferative, cell death-inducing and immunomodulatory effects over IFN-alpha in HTLV-1-associated myelopathy. PLoS Negl. Trop. Dis. 6:e1729 10.1371/journal.pntd.0001729 22848768 PMC3404116

[B123] Morris K. V. Chan S. W. Jacobsen S. E. Looney D. J. (2004). Small interfering RNA-induced transcriptional gene silencing in human cells. Science 305, 1289–1292 10.1126/science.1101372 15297624

[B124] Morris K. V. Santoso S. Turner A. M. Pastori C. Hawkins P. G. (2008). Bidirectional transcription directs both transcriptional gene activation and suppression in human cells. PLoS Genet. 4:e1000258 10.1371/journal.pgen.1000258 19008947 PMC2576438

[B125] Nam E. J. Yoon H. Kim S. W. Kim H. Kim Y. T. Kim J. H. (2008). MicroRNA expression profiles in serous ovarian carcinoma. Clin. Cancer Res. 14, 2690–2695 10.1158/1078-0432.CCR-07-1731 18451233

[B126] Narayanan A. Kehn-Hall K. Bailey C. Kashanchi F. (2011). Analysis of the roles of HIV-derived microRNAs. Expert Opin. Biol. Ther. 11, 17–29 10.1517/14712598.2011.540564 21133815

[B127] Nicolussi E. M. Huck S. Lassmann H. Bradl M. (2009). The cholinergic anti-inflammatory system limits T cell infiltration into the neurodegenerative CNS, but cannot counteract complex CNS inflammation. Neurobiol. Dis. 35, 24–31 10.1016/j.nbd.2009.03.010 19344760

[B128] Nicot C. Mahieux R. Opavsky R. Cereseto A. Wolff L. Brady J. N. (2000). HTLV-I Tax transrepresses the human c-Myb promoter independently of its interaction with CBP or p300. Oncogene 19, 2155–2164 10.1038/sj.onc.1203536 10815807

[B129] Noma K. Sugiyama T. Cam H. Verdel A. Zofall M. Jia S. (2004). RITS acts in cis to promote RNA interference-mediated transcriptional and post-transcriptional silencing. Nat. Genet. 36, 1174–1180 10.1038/ng1452 15475954

[B130] Ooi S. K. Qiu C. Bernstein E. Li K. Jia D. Yang Z. (2007). DNMT3L connects unmethylated lysine 4 of histone H3 to de novo methylation of DNA. Nature 448, 714–717 10.1038/nature05987 17687327 PMC2650820

[B131] Orom U. A. Nielsen F. C. Lund A. H. (2008). MicroRNA-10a binds the 5' UTR of ribosomal protein mRNAs and enhances their translation. Mol. Cell 30, 460–471 10.1016/j.molcel.2008.05.001 18498749

[B132] Ouellet D. L. Plante I. Landry P. Barat C. Janelle M. E. Flamand L. (2008). Identification of functional microRNAs released through asymmetrical processing of HIV-1 TAR element. Nucleic Acids Res. 36, 2353–2365 10.1093/nar/gkn076 18299284 PMC2367715

[B133] Paraboschi E. M. Solda G. Gemmati D. Orioli E. Zeri G. Benedetti M. D. (2011). Genetic association and altered gene expression of mir-155 in multiple sclerosis patients. Int. J. Mol. Sci. 12, 8695–8712 10.3390/ijms12128695 22272099 PMC3257096

[B134] Parker J. S. Parizotto E. A. Wang M. Roe S. M. Barford D. (2009). Enhancement of the seed-target recognition step in RNA silencing by a PIWI/MID domain protein. Mol. Cell 33, 204–214 10.1016/j.molcel.2008.12.012 19187762 PMC2642989

[B135] Parker J. S. Roe S. M. Barford D. (2006). Molecular mechanism of target RNA transcript recognition by Argonaute-guide complexes. Cold Spring Harb. Symp. Quant. Biol. 71, 45–50 10.1101/sqb.2006.71.029 17381279

[B136] Peeters A. Lambert P. F. Deacon N. J. (1996). A fourth Sp1 site in the human immunodeficiency virus type 1 long terminal repeat is essential for negative-sense transcription. J. Virol. 70, 6665–6672 8794302 10.1128/jvi.70.10.6665-6672.1996 PMC190708

[B137] Perez J. T. Varble A. Sachidanandam R. Zlatev I. Manoharan M. Garcia-Sastre A. (2010). Influenza A virus-generated small RNAs regulate the switch from transcription to replication. Proc. Natl. Acad. Sci. U.S.A. 107, 11525–11530 10.1073/pnas.1001984107 20534471 PMC2895093

[B138] Perron M. P. Provost P. (2008). Protein interactions and complexes in human microRNA biogenesis and function. Front. Biosci. 13, 2537–2547 17981733 10.2741/2865 PMC2901379

[B139] Perron M. P. Provost P. (2009). Protein components of the microRNA pathway and human diseases. Methods Mol. Biol. 487, 369–385 10.1007/978-1-60327-547-7_18 19301657 PMC2903565

[B140] Petrocca F. Visone R. Onelli M. R. Shah M. H. Nicoloso M. S. De Martino I. (2008). E2F1-regulated microRNAs impair TGFbeta-dependent cell-cycle arrest and apoptosis in gastric cancer. Cancer Cell 13, 272–286 10.1016/j.ccr.2008.02.013 18328430

[B141] Pfeffer S. Zavolan M. Grasser F. A. Chien M. Russo J. J. Ju J. (2004). Identification of virus-encoded microRNAs. Science 304, 734–736 10.1126/science.1096781 15118162

[B142] Poiesz B. J. Ruscetti F. W. Gazdar A. F. Bunn P. A. Minna J. D. Gallo R. C. (1980). Detection and isolation of type C retrovirus particles from fresh and cultured lymphocytes of a patient with cutaneous T-cell lymphoma. Proc. Natl. Acad. Sci. U.S.A. 77, 7415–7419 6261256 10.1073/pnas.77.12.7415 PMC350514

[B143] Poiesz B. J. Ruscetti F. W. Reitz M. S. Kalyanaraman V. S. Gallo R. C. (1981). Isolation of a new type C retrovirus (HTLV) in primary uncultured cells of a patient with Sezary T-cell leukaemia. Nature 294, 268–271 6272125 10.1038/294268a0

[B144] Pozzatti R. Vogel J. Jay G. (1990). The human T-lymphotropic virus type I tax gene can cooperate with the ras oncogene to induce neoplastic transformation of cells. Mol. Cell. Biol. 10, 413–417 10.1128/MCB.10.1.413 2403646 PMC360770

[B145] Rahman S. Quann K. Pandya D. Singh S. Khan Z. K. Jain P. (2012). HTLV-1 Tax mediated downregulation of miRNAs associated with chromatin remodeling factors in T cells with stably integrated viral promoter. PLoS ONE 7:e34490 10.1371/journal.pone.0034490 22496815 PMC3319589

[B146] Rende F. Cavallari I. Corradin A. Silic-Benussi M. Toulza F. Toffolo G. M. (2011). Kinetics and intracellular compartmentalization of HTLV-1 gene expression: nuclear retention of HBZ mRNAs. Blood 117, 4855–4859 10.1182/blood-2010-11-316463 21398577 PMC5292588

[B147] Reyes J. C. Barra J. Muchardt C. Camus A. Babinet C. Yaniv M. (1998). Altered control of cellular proliferation in the absence of mammalian brahma (SNF2alpha). EMBO J. 17, 6979–6991 10.1093/emboj/17.23.6979 9843504 PMC1171046

[B148] Rigoutsos I. (2009). New tricks for animal microRNAS: targeting of amino acid coding regions at conserved and nonconserved sites. Cancer Res. 69, 3245–3248 10.1158/0008-5472.CAN-09-0352 19351814

[B149] Ruggero K. Corradin A. Zanovello P. Amadori A. Bronte V. Ciminale V. (2010). Role of microRNAs in HTLV-1 infection and transformation. Mol. Aspects Med. 31, 367–382 10.1016/j.mam.2010.05.001 20600265

[B150] Saito M. Matsuzaki T. Satou Y. Yasunaga J. Saito K. Arimura K. (2009). *In vivo* expression of the HBZ gene of HTLV-1 correlates with proviral load, inflammatory markers and disease severity in HTLV-1 associated myelopathy/tropical spastic paraparesis (HAM/TSP). Retrovirology 6:19 10.1186/1742-4690-6-19 19228429 PMC2653460

[B151] Sampey G. Guendel I. Das R. Jaworski E. Klase Z. Narayanan A. (2012). Transcriptional gene silencing (TGS) via the RNAi machinery in HIV-1 infections. Biology 1, 339–369 10.3390/biology1020339 PMC4009781 24832229

[B152] Satou Y. Yasunaga J. Yoshida M. Matsuoka M. (2006). HTLV-I basic leucine zipper factor gene mRNA supports proliferation of adult T cell leukemia cells. Proc. Natl. Acad. Sci. U.S.A. 103, 720–725 10.1073/pnas.0507631103 16407133 PMC1334651

[B153] Schopman N. C. Willemsen M. Liu Y. P. Bradley T. Van Kampen A. Baas F. (2012). Deep sequencing of virus-infected cells reveals HIV-encoded small RNAs. Nucleic Acids Res. 40, 414–427 10.1093/nar/gkr719 21911362 PMC3245934

[B154] Schwarz D. S. Hutvagner G. Du T. Xu Z. Aronin N. Zamore P. D. (2003). Asymmetry in the assembly of the RNAi enzyme complex. Cell 115, 199–208 10.1016/S0092-8674(03)00759-1 14567917

[B155] Selcuklu S. D. Donoghue M. T. Spillane C. (2009). miR-21 as a key regulator of oncogenic processes. Biochem. Soc. Trans. 37, 918–925 10.1042/BST0370918 19614619

[B156] Shaked I. Meerson A. Wolf Y. Avni R. Greenberg D. Gilboa-Geffen A. (2009). MicroRNA-132 potentiates cholinergic anti-inflammatory signaling by targeting acetylcholinesterase. Immunity 31, 965–973 10.1016/j.immuni.2009.09.019 20005135

[B157] Shembade N. Harhaj E. W. (2010). Role of post-translational modifications of HTLV-1 Tax in NF-kappaB activation. World J. Biol. Chem. 1, 13–20 10.4331/wjbc.v1.i1.13 21540989 PMC3083931

[B158] Skalsky R. L. Samols M. A. Plaisance K. B. Boss I. W. Riva A. Lopez M. C. (2007). Kaposi's sarcoma-associated herpesvirus encodes an ortholog of miR-155. J. Virol. 81, 12836–12845 10.1128/JVI.01804-07 17881434 PMC2169101

[B159] Sullivan C. S. Ganem D. (2005). MicroRNAs and viral infection. Mol. Cell 20, 3–7 10.1016/j.molcel.2005.09.012 16209940

[B160] Sun S. C. Yamaoka S. (2005). Activation of NF-kappaB by HTLV-I and implications for cell transformation. Oncogene 24, 5952–5964 10.1038/sj.onc.1208969 16155602

[B161] Suzuki T. Hirai H. Yoshida M. (1994). Tax protein of HTLV-1 interacts with the Rel homology domain of NF-kappa B p65 and c-Rel proteins bound to the NF-kappa B binding site and activates transcription. Oncogene 9, 3099–3105 7936632

[B162] Tagieva N. E. Vaquero C. (1997). Expression of naturally occurring antisense RNA inhibits human immunodeficiency virus type 1 heterologous strain replication. J. Gen. Virol. 78 (Pt 10), 2503–2511 9349471 10.1099/0022-1317-78-10-2503

[B163] Tanaka A. Takahashi C. Yamaoka S. Nosaka T. Maki M. Hatanaka M. (1990). Oncogenic transformation by the tax gene of human T-cell leukemia virus type I *in vitro*. Proc. Natl. Acad. Sci. U.S.A. 87, 1071–1075 2300570 10.1073/pnas.87.3.1071 PMC53412

[B164] Tokudome S. Tokunaga O. Shimamoto Y. Miyamoto Y. Sumida I. Kikuchi M. (1989). Incidence of adult T-cell leukemia/lymphoma among human T-lymphotropic virus type I carriers in Saga, Japan. Cancer Res. 49, 226–228 2908848

[B165] Tomita M. Kikuchi A. Akiyama T. Tanaka Y. Mori N. (2006). Human T-cell leukemia virus type 1 tax dysregulates beta-catenin signaling. J. Virol. 80, 10497–10505 10.1128/JVI.00739-06 16920823 PMC1641756

[B166] Tomita M. Tanaka Y. Mori N. (2012). MicroRNA miR-146a is induced by HTLV-1 tax and increases the growth of HTLV-1-infected T-cells. Int. J. Cancer 130, 2300–2309 10.1002/ijc.25115 20017139

[B167] Trotta R. Chen L. Ciarlariello D. Josyula S. Mao C. Costinean S. (2012). miR-155 regulates IFN-gamma production in natural killer cells. Blood 119, 3478–3485 10.1182/blood-2011-12-398099 22378844 PMC3325038

[B168] Tsukasaki K. Hermine O. Bazarbachi A. Ratner L. Ramos J. C. Harrington W. (2009). Definition, prognostic factors, treatment, and response criteria of adult T-cell leukemia-lymphoma: a proposal from an international consensus meeting. J. Clin. Oncol. 27, 453–459 10.1200/JCO.2008.18.2428 19064971 PMC2737379

[B169] Tsukasaki K. Utsunomiya A. Fukuda H. Shibata T. Fukushima T. Takatsuka Y. (2007). VCAP-AMP-VECP compared with biweekly CHOP for adult T-cell leukemia-lymphoma: Japan Clinical Oncology Group Study JCOG9801. J. Clin. Oncol. 25, 5458–5464 10.1200/JCO.2007.11.9958 17968021

[B170] Uchiyama T. Yodoi J. Sagawa K. Takatsuki K. Uchino H. (1977). Adult T-cell leukemia: clinical and hematologic features of 16 cases. Blood 50, 481–492 301762

[B171] Umbach J. L. Cullen B. R. (2009). The role of RNAi and microRNAs in animal virus replication and antiviral immunity. Genes Dev. 23, 1151–1164 10.1101/gad.1793309 19451215 PMC2763533

[B172] Van Duyne R. Guendel I. Klase Z. Narayanan A. Coley W. Jaworski E. (2012). Localization and sub-cellular shuttling of HTLV-1 Tax with the miRNA machinery. PLoS ONE 7:e40662 10.1371/journal.pone.0040662 22808228 PMC3393700

[B173] Vanhee-Brossollet C. Thoreau H. Serpente N. D'Auriol L. Levy J. P. Vaquero C. (1995). A natural antisense RNA derived from the HIV-1 env gene encodes a protein which is recognized by circulating antibodies of HIV+ individuals. Virology 206, 196–202 10.1016/S0042-6822(95)80034-4 7831774

[B174] Van Wynsberghe P. M. Chan S. P. Slack F. J. Pasquinelli A. E. (2011). Analysis of microRNA expression and function. Methods Cell Biol. 106, 219–252 10.1016/B978-0-12-544172-8.00008-6 22118279 PMC4314212

[B175] Verdel A. Jia S. Gerber S. Sugiyama T. Gygi S. Grewal S. I. (2004). RNAi-mediated targeting of heterochromatin by the RITS complex. Science 303, 672–676 10.1126/science.1093686 14704433 PMC3244756

[B176] Vermeulen A. Behlen L. Reynolds A. Wolfson A. Marshall W. S. Karpilow J. (2005). The contributions of dsRNA structure to Dicer specificity and efficiency. RNA 11, 674–682 10.1261/rna.7272305 15811921 PMC1370754

[B177] Vire E. Brenner C. Deplus R. Blanchon L. Fraga M. Didelot C. (2006). The Polycomb group protein EZH2 directly controls DNA methylation. Nature 439, 871–874 10.1038/nature04431 16357870

[B178] Volinia S. Calin G. A. Liu C. G. Ambs S. Cimmino A. Petrocca F. (2006). A microRNA expression signature of human solid tumors defines cancer gene targets. Proc. Natl. Acad. Sci. U.S.A. 103, 2257–2261 10.1073/pnas.0510565103 16461460 PMC1413718

[B179] Wang D. J. Legesse-Miller A. Johnson E. L. Coller H. A. (2012). Regulation of the let-7a-3 promoter by NF-kappaB. PLoS ONE 7:e31240 10.1371/journal.pone.0031240 22348059 PMC3278432

[B180] Wang H. Yu M. Ochani M. Amella C. A. Tanovic M. Susarla S. (2003). Nicotinic acetylcholine receptor alpha7 subunit is an essential regulator of inflammation. Nature 421, 384–388 10.1038/nature01339 12508119

[B181] Weinberg M. S. Villeneuve L. M. Ehsani A. Amarzguioui M. Aagaard L. Chen Z. X. (2006). The antisense strand of small interfering RNAs directs histone methylation and transcriptional gene silencing in human cells. RNA 12, 256–262 10.1261/rna.2235106 16373483 PMC1370905

[B182] Winter J. Jung S. Keller S. Gregory R. I. Diederichs S. (2009). Many roads to maturity: microRNA biogenesis pathways and their regulation. Nat. Cell Biol. 11, 228–234 10.1038/ncb0309-228 19255566

[B183] Wu K. Bottazzi M. E. De La Fuente C. Deng L. Gitlin S. D. Maddukuri A. (2004). Protein profile of tax-associated complexes. J. Biol. Chem. 279, 495–508 10.1074/jbc.M310069200 14530271

[B184] Wu L. Li H. Jia C. Y. Cheng W. Yu M. Peng M. (2012). MicroRNA-223 regulates FOXO1 expression and cell proliferation. FEBS Lett. 586, 1038–1043 10.1016/j.febslet.2012.02.050 22569260

[B185] Xiao G. Cvijic M. E. Fong A. Harhaj E. W. Uhlik M. T. Waterfield M. (2001). Retroviral oncoprotein Tax induces processing of NF-kappaB2/p100 in T cells: evidence for the involvement of IKKalpha. EMBO J. 20, 6805–6815 10.1093/emboj/20.23.6805 11726516 PMC125766

[B186] Xu J. Wu C. Che X. Wang L. Yu D. Zhang T. (2011). Circulating microRNAs, miR-21, miR-122, and miR-223, in patients with hepatocellular carcinoma or chronic hepatitis. Mol. Carcinog. 50, 136–142 10.1002/mc.20712 21229610

[B187] Yamamoto K. Ishida T. Nakano K. Yamagishi M. Yamochi T. Tanaka Y. (2011). SMYD3 interacts with HTLV-1 Tax and regulates subcellular localization of Tax. Cancer Sci. 102, 260–266 10.1111/j.1349-7006.2010.01752.x 21054678

[B188] Yamano Y. Araya N. Sato T. Utsunomiya A. Azakami K. Hasegawa D. (2009). Abnormally high levels of virus-infected IFN-gamma+ CCR4+ CD4+ CD25+ T cells in a retrovirus-associated neuroinflammatory disorder. PLoS ONE 4:e6517 10.1371/journal.pone.0006517 19654865 PMC2715877

[B189] Yanaihara N. Caplen N. Bowman E. Seike M. Kumamoto K. Yi M. (2006). Unique microRNA molecular profiles in lung cancer diagnosis and prognosis. Cancer Cell 9, 189–198 10.1016/j.ccr.2006.01.025 16530703

[B190] Yang J. S. Lai E. C. (2011). Alternative miRNA biogenesis pathways and the interpretation of core miRNA pathway mutants. Mol. Cell 43, 892–903 10.1016/j.molcel.2011.07.024 21925378 PMC3176435

[B191] Yang L. Kotomura N. Ho Y. K. Zhi H. Bixler S. Schell M. J. (2011a). Complex cell cycle abnormalities caused by human T-lymphotropic virus type 1 Tax. J. Virol. 85, 3001–3009 10.1128/JVI.00086-10 21209109 PMC3067921

[B192] Yang M. Chen J. Su F. Yu B. Lin L. Liu Y. (2011b). Microvesicles secreted by macrophages shuttle invasion-potentiating microRNAs into breast cancer cells. Mol. Cancer 10:117 10.1186/1476-4598-10-117 21939504 PMC3190352

[B193] Yao Y. Suo A. L. Li Z. F. Liu L. Y. Tian T. Ni L. (2009). MicroRNA profiling of human gastric cancer. Mol. Med. Rep. 2, 963–970 10.3892/mmr_00000199 21475928

[B194] Yasunaga J. Matsuoka M. (2011). Molecular mechanisms of HTLV-1 infection and pathogenesis. Int. J. Hematol. 94, 435–442 10.1007/s12185-011-0937-1 21953273

[B195] Yeung M. L. Bennasser Y. Watashi K. Le S. Y. Houzet L. Jeang K. T. (2009). Pyrosequencing of small non-coding RNAs in HIV-1 infected cells: evidence for the processing of a viral-cellular double-stranded RNA hybrid. Nucleic Acids Res. 37, 6575–6586 10.1093/nar/gkp707 19729508 PMC2770672

[B196] Yeung M. L. Yasunaga J. Bennasser Y. Dusetti N. Harris D. Ahmad N. (2008). Roles for microRNAs, miR-93 and miR-130b, and tumor protein 53-induced nuclear protein 1 tumor suppressor in cell growth dysregulation by human T-cell lymphotrophic virus 1. Cancer Res. 68, 8976–8985 10.1158/0008-5472.CAN-08-0769 18974142 PMC2596768

[B197] Yi R. Qin Y. Macara I. G. Cullen B. R. (2003). Exportin-5 mediates the nuclear export of pre-microRNAs and short hairpin RNAs. Genes Dev. 17, 3011–3016 10.1101/gad.1158803 14681208 PMC305252

[B198] Yin M. J. Christerson L. B. Yamamoto Y. Kwak Y. T. Xu S. Mercurio F. (1998). HTLV-I Tax protein binds to MEKK1 to stimulate IkappaB kinase activity and NF-kappaB activation. Cell 93, 875–884 10.1016/S0092-8674(00)81447-6 9630230

[B199] Yin M. J. Paulssen E. J. Seeler J. S. Gaynor R. B. (1995). Protein domains involved in both *in vivo* and *in vitro* interactions between human T-cell leukemia virus type I tax and CREB. J. Virol. 69, 3420–3432 7745688 10.1128/jvi.69.6.3420-3432.1995 PMC189054

[B200] Ying S. Y. Lin S. L. (2009). Intron-mediated RNA interference and microRNA biogenesis. Methods Mol. Biol. 487, 387–413 10.1007/978-1-60327-547-7_19 19301658

[B201] Yoshida M. Miyoshi I. Hinuma Y. (1982). Isolation and characterization of retrovirus from cell lines of human adult T-cell leukemia and its implication in the disease. Proc. Natl. Acad. Sci. U.S.A. 79, 2031–2035 6979048 10.1073/pnas.79.6.2031 PMC346116

[B202] Zhang X. Zeng Y. (2010). The terminal loop region controls microRNA processing by Drosha and Dicer. Nucleic Acids Res. 38, 7689–7697 10.1093/nar/gkq645 20660014 PMC2995066

[B203] Zhao L. J. Giam C. Z. (1992). Human T-cell lymphotropic virus type I (HTLV-I) transcriptional activator, Tax, enhances CREB binding to HTLV-I 21-base-pair repeats by protein-protein interaction. Proc. Natl. Acad. Sci. U.S.A. 89, 7070–7074 1386673 10.1073/pnas.89.15.7070 PMC49647

[B204] Zhao Y. Xu H. Yao Y. Smith L. P. Kgosana L. Green J. (2011). Critical role of the virus-encoded microRNA-155 ortholog in the induction of Marek's disease lymphomas. PLoS Pathog. 7:e1001305 10.1371/journal.ppat.1001305 21383974 PMC3044692

[B205] Zhi H. Yang L. Kuo Y. L. Ho Y. K. Shih H. M. Giam C. Z. (2011). NF-kappaB hyper-activation by HTLV-1 tax induces cellular senescence, but can be alleviated by the viral anti-sense protein HBZ. PLoS Pathog. 7:e1002025 10.1371/journal.ppat.1002025 21552325 PMC3084201

[B206] Zofall M. Grewal S. I. (2006). RNAi-mediated heterochromatin assembly in fission yeast. Cold Spring Harb. Symp. Quant. Biol. 71, 487–496 10.1101/sqb.2006.71.059 17381331

